# Early Human Hemogenic Endothelium Generates Primitive and Definitive Hematopoiesis *In Vitro*

**DOI:** 10.1016/j.stemcr.2018.09.013

**Published:** 2018-10-25

**Authors:** Eva Garcia-Alegria, Sara Menegatti, Muhammad Z.H. Fadlullah, Pablo Menendez, Georges Lacaud, Valerie Kouskoff

**Affiliations:** 1 Developmental Haematopoiesis Group, Faculty of Biology, Medicine and Health, The University of Manchester, Manchester M13 9PT, UK; 2 Stem Cell Biology Group, CRUK Manchester Institute, The University of Manchester, Manchester M20 4BX, UK; 3 Josep Carreras Leukemia Research Institute and Department of Biomedicine, School of Medicine, University of Barcelona, Barcelona, Spain; 4 Instituciò Catalana Recerca i Estudis Avançats (ICREA), 08010 Barcelona, Spain

**Keywords:** hESC, hemogenic endothelium, multilineage potential, definitive hematopoiesis, primitive hematopoiesis

## Abstract

The differentiation of human embryonic stem cells (hESCs) to hematopoietic lineages initiates with the specification of hemogenic endothelium, a transient specialized endothelial precursor of all blood cells. This *in vitro* system provides an invaluable model to dissect the emergence of hematopoiesis in humans. However, the study of hematopoiesis specification is hampered by a lack of consensus in the timing of hemogenic endothelium analysis and the full hematopoietic potential of this population. Here, our data reveal a sharp decline in the hemogenic potential of endothelium populations isolated over the course of hESC differentiation. Furthermore, by tracking the dynamic expression of CD31 and CD235a at the onset of hematopoiesis, we identified three populations of hematopoietic progenitors, representing primitive and definitive subsets that all emerge from the earliest specified hemogenic endothelium. Our data establish that hemogenic endothelium populations endowed with primitive and definitive hematopoietic potential are specified simultaneously from the mesoderm in differentiating hESCs.

## Highlights

• Hemogenic potential decreases along the differentiation timing of hESCs
• Primitive erythrocytes and T cells emerge both from early hemogenic endothelial
• Loss of CD31 expression correlates with restricted hematopoietic potential
• Dynamic expression of CD31 and CD235a defines two waves of human hematopoiesis

## Graphical Abstract

**Figure:**
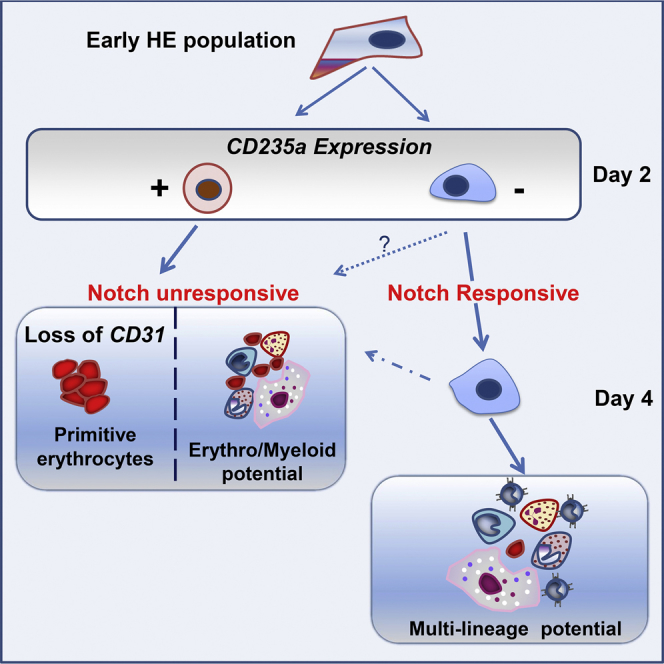

## Teaser

Following the *in vitro* differentiation of human ESCs, Garcia-Alegria et al. demonstrate how the timing of endothelial cell isolation influences hemogenic potential. Early endothelial cells have the highest hemogenic potential, giving rise to two waves of hematopoiesis that are distinguished by CD31/CD235a expression. Multilineage potential is restricted to CD31^+^ cells and commitment toward primitive erythropoiesis coincides with CD31 loss.

## Introduction

The hemogenic endothelium (HE) has been described as an intermediate endothelial precursor of all hematopoietic progenitors in the human embryo (Ivanovs et al., 2017, Julien et al., 2016). The *in vitro* derivation of this specialized endothelium from human embryonic stem cells (hESCs) provides an invaluable platform to study and dissect blood specification and the emergence of hematopoietic stem and progenitor cells. In the last decade, there has been an increased interest in the characterization of this precursor from differentiating hESCs using several approaches, mainly through three-dimensional embryoid body (EB) differentiation (Ditadi et al., 2015, Kennedy et al., 2012, Pick et al., 2007, Ramos-Mejia et al., 2014, Sturgeon et al., 2014), or co-culture on stromal cell lines (Choi et al., 2012, Rafii et al., 2013). The efficiency of hematopoietic differentiation differs between the two methodologies due to parameters such as serum, stromal maintenance, or EB size, among others factors (Kardel and Eaves, 2012, Vodyanik et al., 2006). More importantly, in both of these experimental approaches, the hemogenic potential of endothelium precursor population has been analyzed at different times of the differentiation process, with or without a prior purification step of this population (Ditadi et al., 2015, Ramos-Mejia et al., 2014). Together these variations in experimental approaches make it difficult to reach clear conclusions and consensus about the nature and potential of HE cells. To date, it is still not known whether HE subsets with different hematopoietic potentials emerge in successive waves during the course of hESC differentiation, whether HE populations are maintained within the differentiating culture over time, or whether one unique population of HE is generated early from mesoderm and progressively differentiates within the culture. Following the hemogenic potential of endothelium cell populations continuously over the course of hESC differentiation would address some of these issues but to date this has never been reported.

Despite these outstanding questions, significant advances have been achieved in the characterization of human HE using different culture conditions (Ditadi et al., 2015, Ng et al., 2016, Rafii et al., 2013, Sturgeon et al., 2014). Using OP9 stromal cells to differentiate hESCs, both Rafii et al. (2013) and Choi et al. (2012) showed that lack of CD73 expression marked endothelium with hemogenic potential, while the upregulation of CD73 marked commitment to endothelium devoid of hematopoietic potential. These findings were also reported using the EB differentiation approach by Ditadi et al. (2015), who further distinguished human HE from vascular endothelium by lack of both CD184 arterial marker and DLL4 Notch ligand expression. This Notch ligand was also shown to regulate the hematopoietic fate of human hemato-endothelial progenitors (Ayllon et al., 2015). To date, a consensus on the immuno-phenotype of human HE indicates that this specialized endothelial precursor is contained within a population co-expressing CD31, CD34, VE-cadherin (CD144), and KDR, and lacking the expression of CD43, CD41, and CD45 marking hematopoietic commitment as well as lacking the expression of DLL4, CD73, and CD184, marking further endothelial commitment or arterial specification. To date, a large amount of data detailing the emergence of blood cells from human HE have been obtained using stromal co-culture protocols (Choi et al., 2012, Rafii et al., 2013, Vodyanik et al., 2006). In those cultures, different hematopoietic populations emerged from CD144^+^CD31^+^CD73^−^ endothelial progenitors, with CD43 expression marking the earliest step of hematopoietic commitment (Vodyanik et al., 2006). Using EB differentiation protocols, the onset of hematopoietic commitment was also defined by the expression of CD43, emerging from a CD34^+^ endothelial precursor population (Kennedy et al., 2012). At later EB stage, most CD43^+^ cells upregulated the expression of CD41a and CD235a, and were enriched for megakaryocyte and erythroid progenitors, respectively (Klimchenko et al., 2009, Paluru et al., 2014). Definitive hematopoiesis, defined by T lymphoid potential, was restricted to the CD43^−^ fraction by day 9 of EB differentiation and to the CD43^low^ by day 11 of EB differentiation (Kennedy et al., 2012). In most of these studies, the endothelial precursor population from which hematopoiesis emerged was not purified, making it difficult to dissociate cell-intrinsic effects from microenvironment-induced influences. Despite these significant advances in our understanding of the onset of *in vitro* human hematopoiesis, further delineation of the progressive specification and clonogenicity of emerging blood progenitors is still required to better characterize the full potential of these progenitors and to possibly identify long-term repopulating hematopoietic stem cells.

In the present study, we have analyzed the hemogenic potential of endothelium precursor populations isolated at days 6, 8, and 10 of EB differentiation and showed that this hemogenic potential declines sharply over the course of the differentiation process. By tracking the kinetics of CD31 expression, we were able to monitor closely the generation of three populations of hematopoietic subsets from HE-enriched endothelium isolated at day 6 of EB differentiation: (1) a first population of progenitors that lost CD31 expression rapidly and contained hematopoietic potential restricted to primitive erythrocytes; (2) a second population of erythroid and myeloid progenitors that co-expressed CD31 and CD235a and was unresponsive to Notch inhibition; (3) a third population of multilineage progenitors that expressed CD31, but not CD235a, was responsive to Notch inhibition and gave rise to erythroid, myeloid, and T lymphoid cells.

## Results

### Transcriptional Landscape of Endothelium Precursor Populations during the Course of EB Differentiation

To address how timing of hESC differentiation might affect hemogenic potential, we first analyzed the emergence of endothelium cell populations during EB differentiation. Similar to studies performed on OP9 co-culture (Choi et al., 2012, Rafii et al., 2013), a significant CD31^+^CD144^+^ endothelial population was detected by day 5 of EB differentiation (Figure S1A). The expansion of this endothelial population was highly dependent on the presence of vascular endothelial growth factor A (VEGF-A) (Figure S1A).

**Figure 1:**
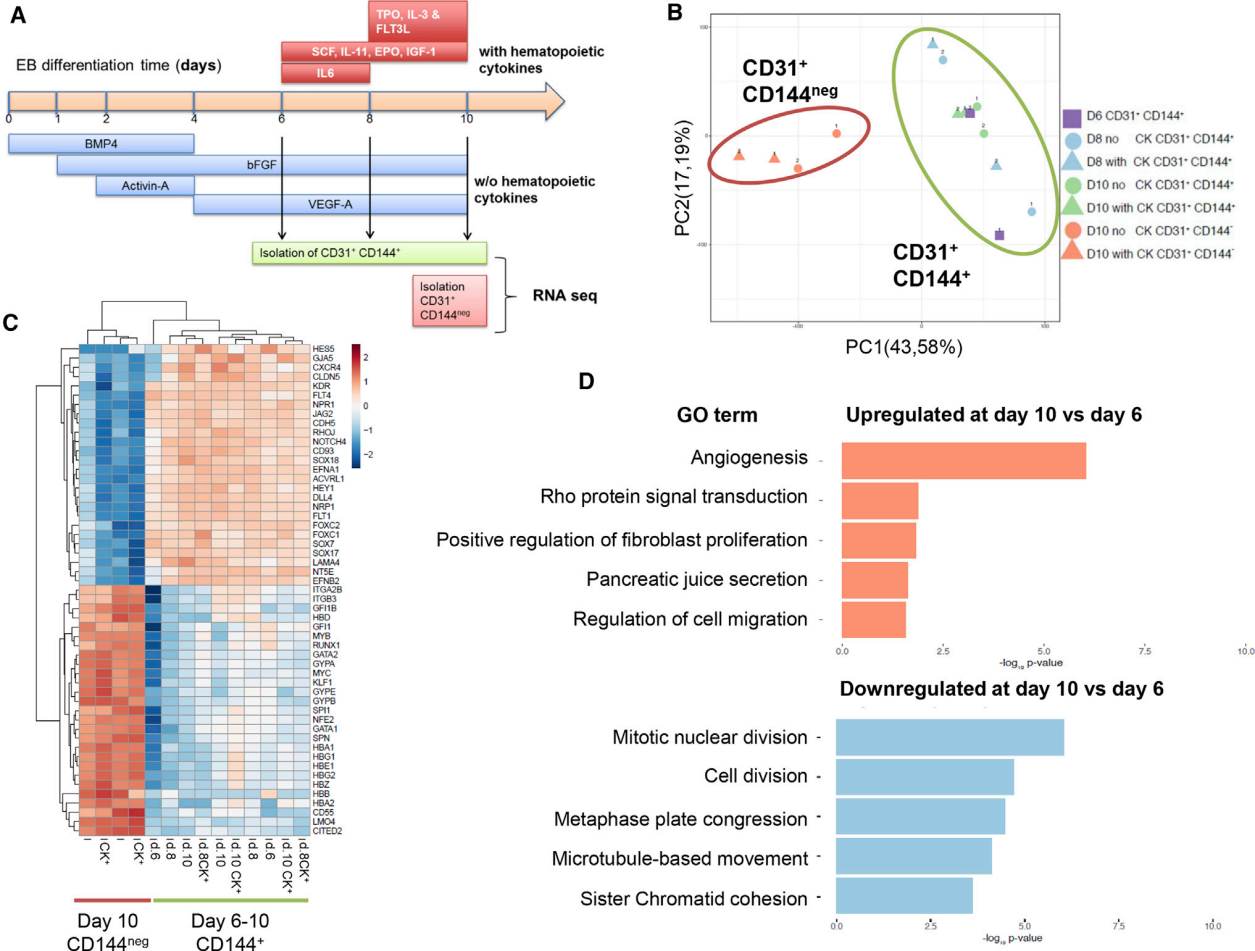
Transcriptional Landscape of CD31^+^CD144^+^ Populations along the EB Differentiation Time (A) Design of the experiment: CD31^+^CD144^+^ populations were isolated along the embryoid body (EB) differentiation timeline with or without the addition of hematopoietic cytokines at the indicated times. CD31^+^CD144^–^ populations were obtained from day 10 of differentiation. (B) PCA plot obtained from the transcriptomic analysis of the CD31^+^CD144^+^ populations isolated at days 6, 8, and 10 of EB differentiation with or without hematopoietic cytokines (CK) added as well as the hematopoietic-committed CD31^+^CD144^–^ populations. (C) Heatmap of representative endothelial and hematopoietic genes differently expressed between CD31^+^CD144^+^ and CD31^+^CD144^–^ populations. (D) Gene ontology (GO) of biological process terms enriched or downregulated in connection with the timing of CD31^+^CD144^+^ isolation were identified using the annotation on line tool DAVID. Data are based on the DEGs obtained between days 6 and 10 of EB differentiation.

As a first approach to assess differences, we compared the transcriptomes of endothelial cell populations isolated at successive time points of differentiation. Cell populations expressing CD31 and CD144 were isolated by fluorescence-activated cell sorting (FACS) at days 6, 8, and 10 of EB differentiation (Figure 1A). At days 8 and 10 of differentiation, CD31^+^CD144^+^ cell populations were isolated from EB cultures supplemented or not with hematopoietic cytokines from day 6 onward. In addition, CD31^+^CD144^−^ cell populations, which have downregulated CD144 expression and are committed to hematopoiesis, were isolated from day 10 EB cultures as a reference for the hematopoietic transcriptomic landscape. All populations were isolated from two independent EB differentiation cultures, and all transcriptomes were determined by RNA sequencing (RNA-seq). Analysis of these datasets by principal-component analysis (PCA) revealed that CD31^+^CD144^+^ subsets did not cluster according to their day of isolation but rather homogenously together away from the CD31^+^CD144^−^ hematopoietic-committed cells (Figure 1B). In line with the PCA results, few genes were found differentially expressed between the various populations (Table S1). No genes were found significantly differentially expressed between days 6 and 8, and only three genes between days 8 and 10. In contrast, 172 genes were found significantly differentially expressed between days 6 and 10, 71 of which were upregulated and 101 downregulated in the day 10 population (Table S1). The addition of cytokines to the EB cultures had little incidence on the transcriptome of the CD31^+^CD144^+^ populations since 0 and 12 genes were found differentially expressed at days 8 and 10, respectively. In contrast, comparison with the CD31^+^CD144^−^ committed hematopoietic cells revealed 3,838 genes differentially expressed, with 1,567 genes upregulated and 2,271 genes downregulated in the CD144^–^ population (Figure S1B). Within these differentially expressed genes (DEGs), critical hematopoietic genes were upregulated and critical endothelial genes were downregulated (Figure 1C) in the CD31^+^CD144^−^ cell populations. Despite the limited transcriptional differences found among the CD31^+^CD144^+^ populations, gene ontology analysis of the DEGs found between days 6 and 10 revealed that the most upregulated process was angiogenesis (Figure 1D). The transcriptomic data indicated an increase in the expression of proangiogenic genes, such as Notch ligand *DLL4* and *CD93,* in the day 10 populations; these observations were further validated by flow cytometry (Figure S2A). In contrast, the most downregulated gene ontology terms were linked to cell division (Figure 1D), an observation that was further validated by cell-cycle analysis of the isolated populations (Figure S2B) and revealed an increased frequency of cells in the G0/G1 phase along with a significant decreased frequency of cells in the G2/M phase in CD31^+^CD144^+^ populations at days 8 and 10 of differentiation.

Together, this transcriptomic analysis revealed little differences in overall gene expression in the endothelial cell populations across the timing of EB differentiation, suggesting the maintenance of the CD31^+^CD144^+^ population within the EB microenvironment.

### Hemogenic Potential of Endothelium Precursor Populations during the Course of EB Differentiation

**Figure 2:**
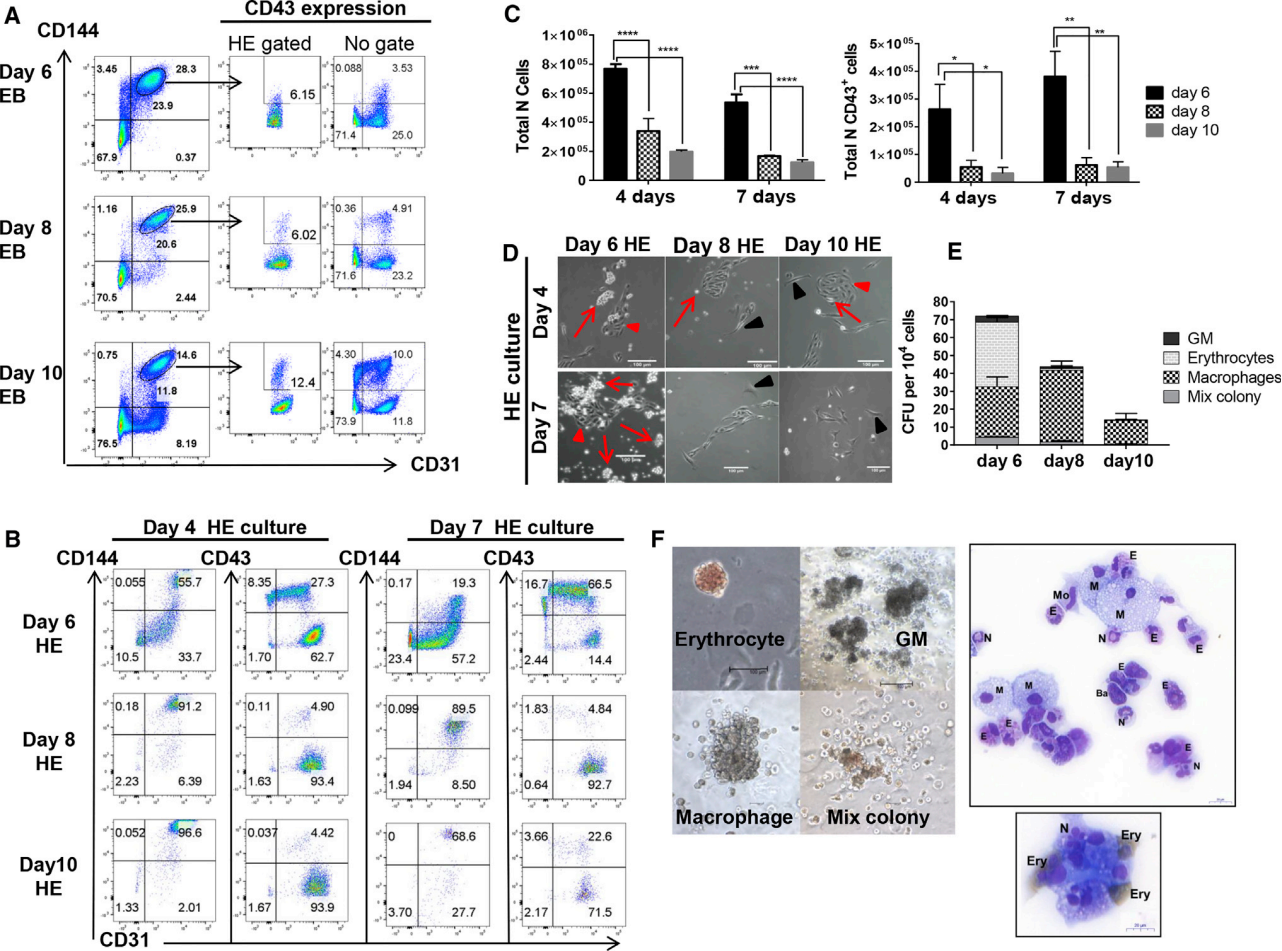
Characterization of Human HE along Human EB Differentiation (A) Flow cytometry analysis of CD31^+^CD144^+^ populations at different times of EB differentiation (representative plots from four different experiments). Circled gates indicate the HE-enriched isolated populations along with the percentage of CD43^+^ cells found within these gated populations or without gating. CD31^+^CD144^+^CD43^+^ cells were excluded during the sort. (B) Kinetic of endothelial (CD31^+^CD144^+^) and hematopoietic (CD43^+^) profiles obtained after re-plating in hematopoietic condition the purified CD31^+^CD144^+^ populations obtained at days 6, 8, and 10 of EB differentiation. Data are representative of three independent experiments after 4 and 7 days in culture on gelatin-coated plates. (C) Quantification and statistical analysis of the total number of cells and hematopoietic-committed cells (CD43^+^) generated at days 4 and 7 of culture. Error bars indicate the SEM of data from three independent experiments. The significance of the difference between samples was confirmed using two-way ANOVA; p value: adjusted p value using SIDAK multiple comparison: ^∗^p < 0.05, ^∗∗^p < 0.001, ^∗∗∗^p = 0.0002, ^∗∗∗∗^p < 0.0001. (D) Representative pictures of CD31^+^CD144^+^ populations isolated at the indicated times of EB differentiation after 4 and 7 days of culture on gelatin-coated plates in hematopoietic-inducing condition. Red arrows indicate endothelial clusters with hemogenic ability. Red arrowheads indicate emerging blood cells and clusters of blood cells. Black arrowheads indicate endothelial cell clusters with no hemogenic potential. (E) Quantification of colony-forming unit (CFU) potential of 10^4^ cells harvested after 4 days of culture from the CD31^+^CD144^+^ populations isolated at days 6, 8, and 10 of EB differentiation. Error bars indicate the SEM of data from three independent experiments. (F) Pictures of representative colonies and cytospin of cells stained with O-dianisidine MGG. Scale bars, 100 μm. M, macrophages; N, neutrophils; E, eosinophils; Ba, basophils; Mo, monocytes; Ery, erythrocytes. Brown staining is indicative of hemoglobinization of the erythrocytes. Scale bars, 20 μm.

Given the low level of changes observed in the transcriptional landscape, we next examined whether the hemogenic potential of the CD31^+^CD144^+^ populations remained constant over the time course of EB differentiation. To address this, CD31^+^CD144^+^CD43^−^ cell populations were isolated by FACS at days 6, 8, and 10 of EB differentiation (Figure 2A) and seeded on gelatin-coated dishes in serum-free culture supplemented with hematopoietic cytokines. Exclusion of CD43-expressing cells ensured that we did not include cells already committed to hematopoiesis in the assay. The immuno-phenotype of cells generated within these hematopoietic-inducing cultures were then analyzed by flow cytometry at days 4 and 7. Hematopoiesis emergence was assessed via the upregulation of CD43 expression, which marks commitment to blood lineages (Kennedy et al., 2012, Vodyanik et al., 2006). Concomitant to CD43 upregulation, CD144 expression was downregulated while CD31 expression was retained on most CD43^+^ cells (Figure 2B). The percentage of CD43^+^ cells produced after 4 and 7 days was very high (over 70%) in cultures initiated with CD31^+^CD144^+^ cells isolated from day 6 EBs. In contrast, the percentage of CD43^+^ cells decreased sharply in cultures initiated from day 8 and 10 EB cell populations. The same trend was observed when comparing the absolute number of CD43^+^ cells harvested from each culture (Figure 2C). Analysis of other hematopoietic markers (CD71, CD41a, and CD61) revealed similar findings (Figure S2C). Maintenance of an endothelial immuno-phenotype was monitored by co-expression of both CD144 and CD31. The frequency of CD31^+^CD144^+^ endothelial cells was inversely correlated to the frequency of CD43^+^ cells (Figure 2B). Cultures initiated from day 8 and 10 EB cells contained increasing frequencies of endothelial cells with low proliferative potential as reflected by the low absolute number of cells obtained from these cultures (Figure 2C). In line with this immuno-phenotypic characterization, the morphology of the cells in cultures initiated from day 6 EB populations showed well-defined clusters of endothelial cells undergoing endothelial-to-hematopoietic transition surrounded by hematopoietic clusters (Figure 2D). These clusters were rarely observed in cultures initiated from day 8 and 10 EB cell populations in which endothelial-type clusters were mainly observed. Finally, clonogenic potential analysis at day 4 of the hematopoietic-inducing cultures further demonstrated the higher hemogenic potential of endothelial precursors isolated from day 6 EBs with a progressive decline in clonogenic potential in cultures derived from CD31^+^CD144^+^ isolated from day 8 and 10 EBs (Figure 2E). By day 7 of hematopoietic-inducing culture, multilineage potential was only maintained in cultures derived from CD31^+^CD144^+^ isolated from day 6 EBs (Figure S2D). May-Grünwald Giemsa O-dianisidine staining of cells from colonies obtained from day 6-derived cultures showed a broad range of blood cell types including monocytes, basophils, and neutrophils, as well as primitive erythrocytes seen as large, nucleated, and stained brown by O-dianisidine (Figure 2F).

Having defined the optimal timing for analyzing HE potential, we next compared the progression of HE culture with or without a purification step of the CD31^+^CD144^+^ population at day 6 of EB differentiation (Figure S3A). We observed considerable outgrowth of mesenchymal-like cells when the whole EB population was seeded (Figures S3B–S3D). This, together with the lower hematopoietic cells output observed without sorting (Figures S3B–S3D) led us to include a sorting step of the CD31^+^CD144^+^ population in the subsequent analysis of hematopoietic specification.

In addition, we further characterized the immuno-phenotype of the hemogenic-enriched endothelial population at day 6 of EB differentiation. Previous studies have described this population as CD31^+^CD144^+^ co-expressing KDR and CD34 but lacking the expression of the hematopoietic markers CD41, CD43, and the expression of the endothelial markers CD73, DLL4, and CXCR4 (Choi et al., 2012, Ditadi et al., 2015). We confirmed this immuno-phenotype using three different hESC lines and further characterized this cell population as expressing CD151 and CD44 (Figures S4A and S5A). Around 5%–15% of the CD144^+^CD31^+^ population did not express CD44, and we therefore analyzed the hemogenic potential of both CD44^−^ and CD44^+^ subsets. We observed a significant increase in the hematopoietic potential of the population expressing CD44 (Figure S4B), confirming the expression of CD44 by HE. These markers, although not previously described in HE, are related to the angiogenic properties of endothelial cells (Cao et al., 2006) and were shown to be expressed by CD34 cord blood cells with hemogenic properties (Pelosi et al., 2012). Furthermore, these cell surface molecules have also been described in the context of adhesion and migration processes (Savani et al., 2001), reinforcing their relevance at the onset of the transition from HE to blood cells (Lie et al., 2014).

Altogether our data demonstrate that the hemogenic potential of the endothelial precursor population in differentiating EBs declines over time. Our findings suggest that the earliest endothelial precursor population is enriched in HE and that these HE cells may mature within the EBs upon further differentiation. In line with our transcriptome analysis, the proliferative potential of endothelium cell population progressively decreases while these cells increase their endothelial identity.

### Hematopoietic Progression from Early HE-Enriched Population

We next aimed to further characterize the onset of hematopoietic progenitor emergence from flow cytometry-enriched HE cells. CD31^+^CD144^+^CD43^−^ cells were isolated from day 6 EBs and further cultured in hematopoietic-inducing condition on gelatin-coated plate or on OP9 stroma cells expressing or not the human Notch ligand DLL1. These stroma lines were included in the experimental design to assess whether stroma interaction might influence the outcome of the culture.

**Figure 3:**
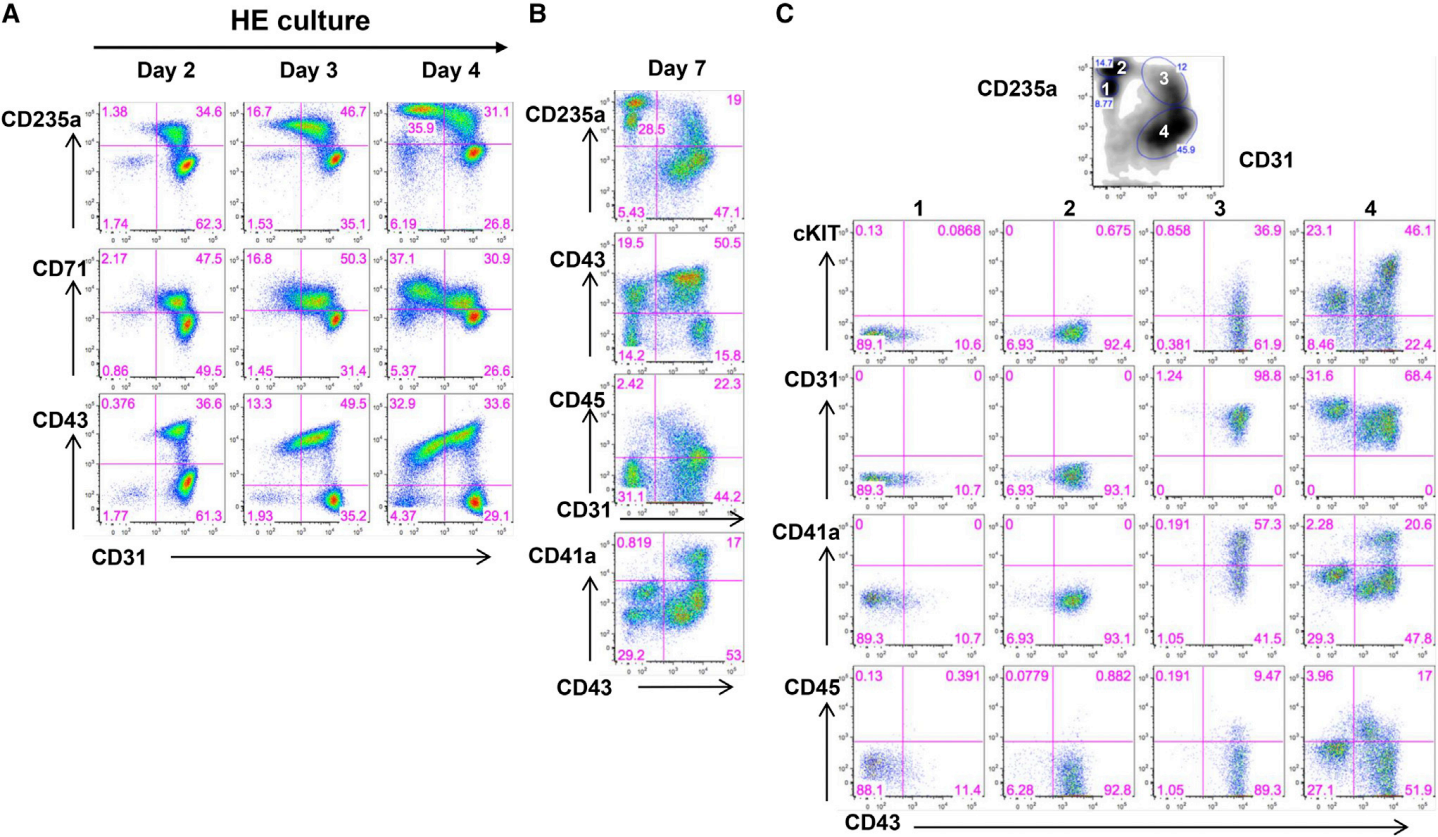
Hematopoietic Progression from EB Day 6-Sorted HE-Enriched Population (A) Flow cytometric analysis showing the immuno-phenotype of hematopoietic populations obtained at days 2, 3, and 4 of the culture of CD144^+^CD31^+^ cells on gelatin-coated plates in hematopoietic-inducing conditions. Data are representative of five independent experiments. (B) Progression of the immuno-phenotype of hematopoietic populations obtained after 7 days of culture on gelatin-coated plates. Data are representative of five independent experiments. (C) Phenotypic characterization of the different cell populations defined by CD235a and CD31 expression after 7 days of HE culture on gelatin. Data are representative of five independent experiments.

In line with published data, the first hematopoietic cells observed in the cultures performed on gelatin expressed the hematopoietic markers CD43 and CD235a (Vodyanik et al., 2006). By day 2 of culture, all CD43^+^ and CD235a^+^ cells still co-expressed CD31 along with CD71 and CD61 (Figures 3A and S4C), but had already downregulated KDR and CD144 (Figure S4D). In these cultures, most of the early emerging hematopoietic cells co-expressed CD41a (Figure S4C), suggesting that in human hematopoiesis the earliest blood cells derived from HE express CD41, similar to what was observed during the emergence of the murine hematopoietic system (Ferkowicz et al., 2003, Mikkola et al., 2003). The kinetics of expression of CD235a, CD71, and CD43 over time in the culture revealed an expansion of these cells accompanied by a progressive loss of CD31-only-expressing cells (Figure 3A). However, a subset of CD31^+^ cells, negative for all hematopoietic markers tested, remained up to day 4 of the culture (Figure 3A). The hematopoietic markers CD61 and CD41a were most exclusively expressed within the CD43^+^CD31^+^ population, representing around 20% of the hematopoietic cells by day 4 of culture (Figure S4C). By day 7 of the culture, 70% of the cells expressed the hematopoietic marker CD43 with a subset co-expressing the pan hematopoietic marker CD45; most CD45^+^ cells co-expressed CD31 (Figure 3B). A similar progression of these hematopoietic subsets was observed upon isolation and further culture of the CD31^+^CD144^+^ populations from two other differentiating hESC lines (Figure S5B).

Using the dynamic expression of CD235a and CD31, four populations were identified both on gelatin (Figures 3B and 3C) and on stromal co-culture (Figure S6A). These four populations were further characterized at day 7 of culture for co-expression of other cell surface markers (Figures 3C, S6A, and S6B). A small population within the CD235a^+^CD31^−^ fraction (population 1) was characterized by lower CD235a expression and loss of CD43 expression. This population together with the CD235a^+^CD43^+^CD31^−^ population (population 2) did not express c-KIT, CD41a, or CD45. In contrast, the two CD31^+^ populations (populations 3 and 4) contained all the CD43^+^CD41a^+^ progenitors and were enriched for c-KIT expression known to mark blood progenitors. The main differences in population 4 compared with population 3 were (1) a lack of CD235a expression, (2) higher frequency of CD45^+^ cells, and (3) lower frequency of CD41a^+^ cells. Similar findings were observed when HE-enriched cells from day 6 EBs were co-cultured on OP9 or OP9 hDLL1 (Figure S6A). A summary of the markers expressed by the different populations at day 7 is shown in Figure S6B.

Together, this analysis further refines our understanding of the immuno-phenotype and dynamic of the very first steps of hematopoietic specification from HE-enriched populations.

### The Multilineage Potential of CD31^+^ Cells Is Maintained on Stroma Co-culture

**Figure 4:**
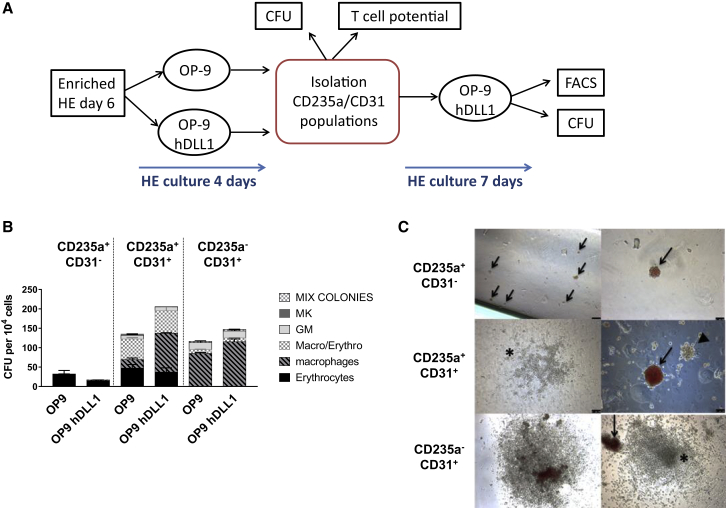
Hematopoietic Potential of CD235a/CD31 Populations (A) Schematic of the experimental design. HE-enriched cells were isolated at day 6 of EB differentiation and co-cultured on OP9 or OP9-hDLL1 stroma for 4 days. At this time point, based on their expression of CD235a and CD31, three populations were isolated and their clonogenic and T cell potential was tested. Further co-culture of each isolated population was carried on OP9-hDLL1 during 7 days, at which time further flow cytometry analysis and CFU assays were performed. (B) Clonogenic potential obtained from the CD235a/CD31 populations isolated after 4 days of co-culture OP9 or OP9-hDLL1 on stroma cell. Error bars indicate the SEM of data from three independent experiments. (C) Representative pictures of hematopoietic colonies obtained from the indicated populations after 14 days in clonogenic assay (n = 3). Black arrows indicate erythroid colonies; arrowhead indicates macrophage colony and asterisks indicate granulo-macrophage colonies. Scale bars, 100 μm.

The data presented above showed that a CD31-expressing subset that did not express CD235a was still present by day 4 of the culture. This population might represent endothelium lacking hemogenic potential, a hemogenic subtype maintained within the culture, committed hematopoietic cells lacking the expression of CD235a or a mixture of all these. To explore the nature of the CD31^+^CD235a^−^ subset and to determine the biological potential of the other populations defined by CD235a and CD31 expression, we analyzed their output either in a clonogenic assay or upon further culture on stroma cells. Given the very similar profiles obtained for CD235a and CD31 expression in the different culture conditions, further assays were performed with populations isolated after 4 days of culture on OP9 stroma expressing or not hDLL1 since the presence of this supportive niche has been shown to enhance hematopoietic output (Ji et al., 2008). HE-enriched CD31^+^CD144^+^CD43^−^ cells isolated from day 6 EBs were cultured on OP9 stroma and, after 4 days of culture, the three main populations based on CD235a and CD31 expression were isolated and either tested in clonogenic assay or further cultured on OP9-hDLL1 (Figure 4A).

Quantification of the colony-forming unit (CFU) potential by clonogenic assay after 4 days of HE culture revealed that only the CD31^+^ subsets (populations 3 and 4) contained multilineage potential (Figures 4B and 4C). The CD235a^+^CD31^−^ population showed limited clonogenic potential, mostly restricted to typical small primitive erythroid colonies (Figure 4C), consistent with the immuno-phenotype described above. Similar results were observed in cells derived from co-cultures on stroma expressing or not hDLL1 (Figure 4B). The two populations expressing CD31 showed some differences in CFU output, with higher erythroid and macrophage-erythroid potential but lower myeloid potential from cells co-expressing CD235a (Figure 4B).

**Figure 5:**
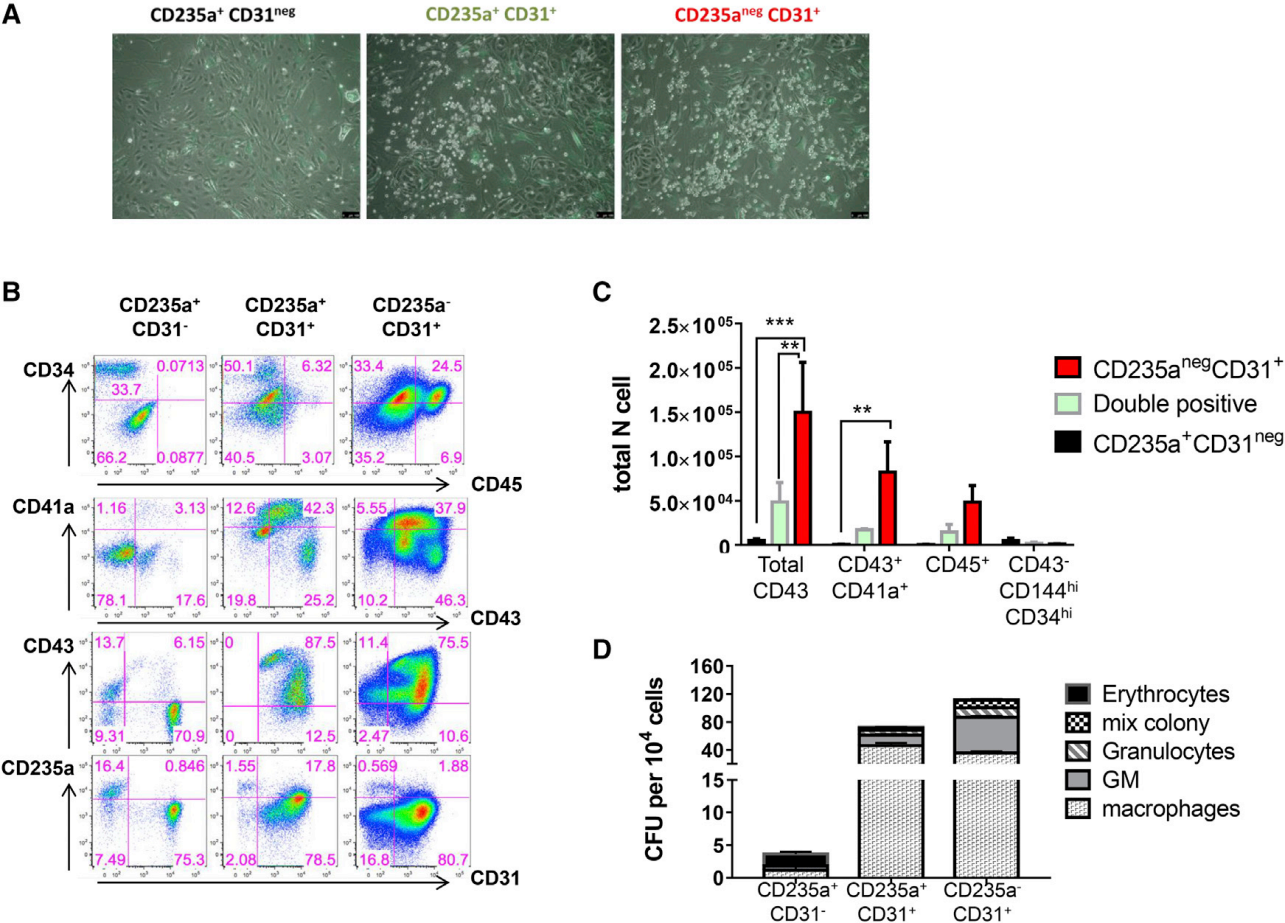
The Multilineage Potential of CD31^+^ Cells Is Maintained on Stroma Co-culture (A) Representative endothelial and hematopoietic cell morphologies displayed by the different CD235a/CD31 populations after purification and co-culture on OP9-hDLL1 stroma cells (n = 3). Scale bars, 100 μm. (B) Representative flow cytometric analysis of the indicated populations after 7 days of culture on OP9-hDLL1 stroma cells (n = 3). (C) Quantification and statistical analysis of the total number of cells obtained after 7 days of culture. Differences between each population were analyzed by two-way ANOVA. Error bars indicate the SEM from three independent experiments; adjusted p values using Turkey multiple comparison test: ^∗∗^p < 0.002, ^∗∗∗^p < 0.0001. (D) Quantification of CFU potential of 10^4^ cells obtained after 7 days of culture from the indicated populations. Error bars indicate the SEM of data from three independent experiments.

In parallel to these clonogenic assays, further co-culture of the isolated CD31/CD235a populations were performed to assess both endothelial and hematopoietic potential and to determine whether co-culture on a supportive niche stroma may enhance or maintain hematopoietic potential. Analysis of endothelial output revealed that a fraction of cells generated from the CD235a^+^CD31^−^ population was endothelial as demonstrated by their morphology (Figure 5A) and immuno-phenotype (Figure 5B). These cells were characterized by CD31 and CD34 expression and lacked hematopoietic marker expression. However, the absolute number of these cells was very low compared with the overall output of cells obtained from the CD31-expressing populations (Figure 5C). Interestingly, when the CD235a^+^CD31^−^ population was further cultured on gelatin rather than on stroma cells, this ability to generate endothelium was not observed (Figure S7A), suggesting that the endothelial potential of the CD235a^+^CD31^–^ population may require a supportive niche to develop. The generation of endothelial cells was barely observed in cultures derived from the CD31-expressing populations (Figures 5B and 5C).

Hematopoietic output was higher in cultures derived from the two CD31^+^ populations (Figures 5A–5D). Most hematopoietic cells derived from the CD235a^+^CD31^−^ population were erythrocytes (Figure 5D); they expressed low levels of CD43 and lacked CD45, CD41a, or CD31 expression (Figure 5B). In line with the clonogenic assay, this population did not give rise to blood progenitors defined as CD43^+^CD34^+^ or CD45^+^CD34^+^, as well as CD43^+^CD41a^+^ megakaryocyte progenitors (Figures 5B and 5C). CD235a expression was mostly observed on cells derived from the CD31^−^CD235a^+^ population. In contrast to the CD31^−^CD235a^+^ population and in line with the clonogenic data, both CD31^+^ populations displayed high hematopoietic output, with most CD43^+^ cells expressing CD34 and maintaining CD31 expression (Figure 5B). However, the CD31^+^CD235a^−^ population generated higher CD45^+^CD34^+^ and CD43^+^CD34^+^ frequencies (Figure 5B). Finally, the clonogenic potential obtained from each of these secondary cultures at day 7 revealed that multilineage potential was only maintained in the two CD31-expressing populations (Figure 5D). These data revealed that this potential is uniquely linked to CD31 expression and can be maintained by co-culture on stroma. Low erythroid-only potential was observed after further culture on stroma and this potential was restricted to the CD235a^+^CD31^–^ population (Figure 5D). Similar hematopoietic differentiation profiles were observed whether HE isolated from day 6 EBs were initially cultured on OP9 (Figure 5B) or OP9 hDLL1 (Figure S6C). Interestingly, only the CD31^+^CD235a^−^ population was affected by the presence of the Notch ligand, resulting in the generation of a greater number of hematopoietic cells when plated on OP9-hDLL1. To determine if the CD31^+^CD235a^−^ population was specifically responsive to the Notch pathway, isolated CD235a/CD31 populations were cultured on OP9-hDLL1 for 7 days with or without the addition of the gamma-secretase inhibitor RO4929097. We observed that only the hematopoietic output obtained from the CD31^+^CD235a^−^ population was affected by Notch signaling inhibition, resulting in an important decrease in the percentage of CD43^+^ and CD43^+^CD41a^+^ cells (Figure S7B). Notch inhibition increased the proportion of cells expressing endothelial markers in all the cultures (Figure S7B).

**Figure 6:**
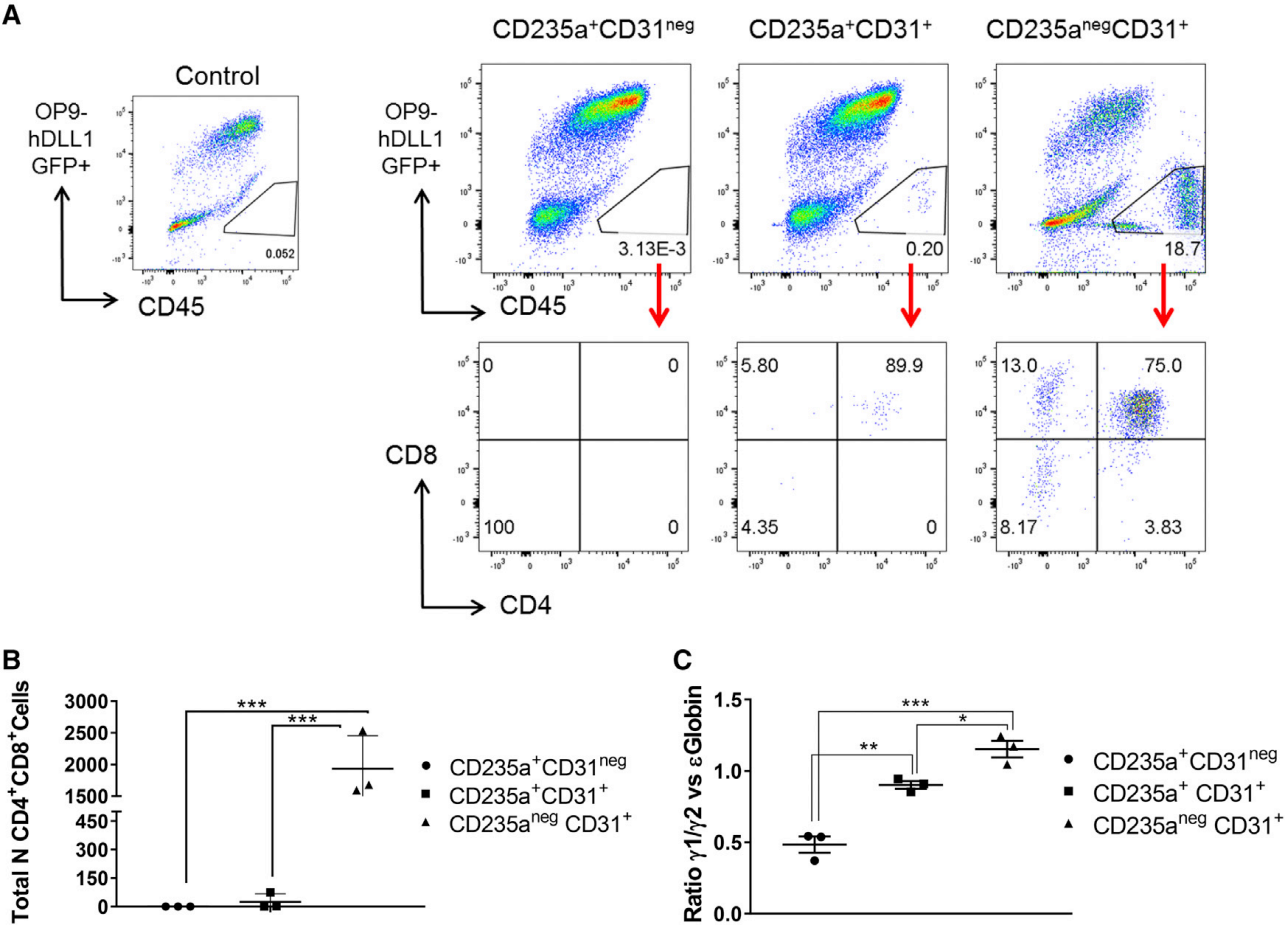
Characterization of Definitive Hematopoietic Potential of the CD31/CD235a Populations (A) T lymphoid potential displayed by the indicated populations after culture on OP9-DLL1. After 1 month, cells were harvested and stained for CD45, CD4, and CD8 expression. Flow cytometric analyses are representative of three independent experiments. Stroma cells were excluded based on their high GFP expression as seen on the control OP9-only culture dot plot on the right. (B) Quantification and statistical analysis of the total number of lymphoid cells obtained in each experiment. Differences between each population were analyzed by two-way ANOVA. Error bars indicate the SEM from three independent experiments. Adjusted p value using Turkey multiple comparison test: ^∗∗∗^p ≤ 0.0006. (C) Globin expression in cells of hematopoietic colonies obtained from the indicated populations after 2 weeks in methylcellulose culture. Quantification and statistical analysis of the ratio between gamma γ1/2 and epsilon ɛ globin expression analyzed by RT-PCR. Error bars indicate the SEM from three independent experiments. Adjusted p value using SIDAK's multiple comparison test: ^∗^p = 0.0326, ^∗∗^p = 0.0028, ^∗∗∗^p = 0.0002.

Given the erythroid-myeloid multilineage potential observed upon clonogenic assay, we next investigate the T lymphoid potential of the three CD235a/CD31 populations obtained from day 4 HE culture (Figure 4A). To test for this potential, the three populations were serially passaged over 1 month on OP9-hDLL1 and then analyzed by flow cytometry. No CD45^+^ cells were found in the cultures derived from the CD235a^+^CD31^−^ population (Figures 6A and 6B). The CD235a^+^CD31^+^ population gave rise to very few CD4^+^CD8^+^ cells, in only one out of three experiments. In sharp contrast, high numbers of T cells expressing CD4 and CD8 were generated from the CD235a^−^CD31^+^ population (Figures 6A and 6B), demonstrating that only this later population is endowed with lymphoid potential, a characteristic of definitive hematopoiesis.

Another characteristic indicative of a shift from primitive to definitive potential is the change in globin gene expression in erythrocytes from ɛ embryonic or primitive globin to γ1/2 fetal globin (Qiu et al., 2008). To further assess the definitive potential of the CD235a^−^CD31^+^ population, we compared the ratio of γ1/2 versus ɛ globin expression in hematopoietic colonies obtained in clonogenic assays performed with the three CD235a/CD31 populations isolated at day 4 of HE culture. A significant increase in the ratio of fetal versus embryonic globin was observed in the CD235a^−^CD31^+^ population compared with the two other populations (Figure 6C), further supporting the definitive hematopoietic potential of the CD235a^−^CD31^+^ population.

**Figure 7:**
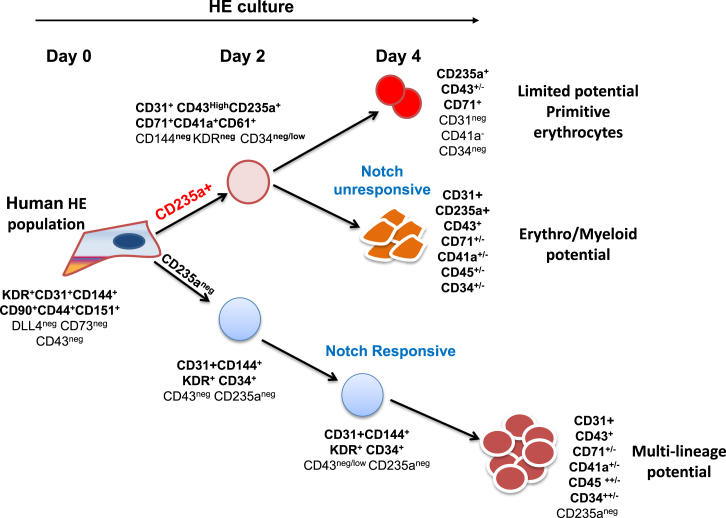
Simultaneous Waves of Hematopoietic Differentiation from HE-Enriched Cell Population Hematopoietic differentiation from HE isolated at day 6 of differentiation. The first hematopoietic wave differentiates rapidly, is detected within 2 days of culture and is characterized by the expression of the hematopoietic marker CD235a and the loss of CD144 and KDR, endothelial and mesodermal markers, respectively (all positive markers in bold characters). From this first wave, two hematopoietic populations emerge by day 4 and are discriminated by CD31 expression. Loss of this marker is accompanied by a reduced clonogenic potential and restriction to primitive erythrocytes. A second wave of hematopoiesis emerging from the same HE-enriched population does not express CD235a and retains endothelial marker expression. This population does not generate CD235a^+^ cells, but gives rise to large numbers of CD45^+^ cells and T cells in culture. The hematopoietic populations generated from this second wave present multilineage potential and are Notch responsive.

Together, these results establish that the expression of CD31 and CD235a define three subsets of blood progenitors that all derive from HE isolated early at day 6 of EB differentiation (Figure 7). Furthermore, these data revealed that high proliferative capacity, multilineage potential and definitive hematopoiesis is closely correlated with CD31 expression and lack of CD235a.

## Discussion

In the present study, we establish that, during the course of human ESC differentiation, an early endothelial precursor population developing from mesoderm initially harbors a high and diverse hemogenic potential, and that this potential declines over time within the differentiating EBs. These findings are consistent with the timeline of hematopoietic development described previously (Kennedy et al., 2012, Rafii et al., 2013, Ramos-Mejia et al., 2014, Sturgeon et al., 2014, Wang et al., 2004) and suggest that, upon ESC differentiation, only one main wave of HE specification from the mesoderm occurs, similar to what has been observed in differentiating mouse ESCs (Pearson et al., 2015).

Establishment of the hematopoietic system during embryonic development is an intricate process involving multiple incremental steps of commitment that are orchestrated by complex networks of transcription factors (Goode et al., 2016, Lacaud and Kouskoff, 2017). The use of mouse and human ESC differentiation as model systems has already allowed unraveling critical aspects of this developmental progression. However, many questions are still outstanding, such as whether HE subsets with specific potential are generated from differentiating ESCs sequentially overtime? To address this important question, we analyzed the transcriptome and hemogenic potential of endothelium populations isolated at days 6, 8, and 10 of ESC differentiation, the timing most commonly used in the published literature (Choi et al., 2012, Kennedy et al., 2012, Real et al., 2012). The overall transcriptional signature observed in these populations was quite homogeneous along the timeline of differentiation and was independent of the addition of hematopoietic cytokines. Differences were observed in the expression levels of genes involved in cell division and angiogenic process, consistent with the observed decrease in proliferative potential of these populations over time and increase endothelial identity. When the hemogenic potential of these successive endothelial populations was determined, a sharp decrease in the quantitative output of blood cell production was observed, with day 8 and 10 populations harboring only minimal hemogenic potential. Importantly, clonogenic assay or immuno-phenotypic characterization did not highlight specific qualitative differences in the output of these three populations except for a sharp decrease in primitive erythrocyte generation. Although we did not test the T cell potential of the day 8 and 10 populations, which could be equivalent or higher than at day 6, it still remains that both erythroid and myeloid potentials were strongly diminished over time. This lack of correlation between transcriptome and changes in hemogenic potential might be explained by a low frequency of HE within these endothelial populations, which therefore contribute little to the overall transcriptomic signature in either population. Alternatively, it is possible that, at the transcriptional level, endothelium precursors endowed with hemogenic potential are extremely similar to non-HE and therefore no differences in transcriptomic landscape are seen whether the endothelial population is hemogenic or not. To date, there are still no cell surface markers identified that are uniquely expressed by HE and that would allow the isolation of a pure HE population.

In this study, we also followed the earliest step of hematopoietic specification from the day 6 HE-enriched population using multi-parameter cell-surface staining, clonogenic assays, and secondary culture of isolated subsets. These experiments established that three populations of hematopoietic progenitors were specified from early HE (Figure 7). The first two subsets both expressed CD235a and CD43, but were distinguished by CD31 expression: the CD235a^+^CD43^+^CD31^−^ population had restricted proliferation and only generated primitive erythroid colonies; the CD235a^+^CD43^+^CD31^+^ population had higher proliferative potential and gave rise to multilineage erythroid and myeloid colonies. These two subsets no longer expressed KDR and CD144 and were fully committed to hematopoiesis as early as 2 days after the initiation of culture. The third population did not initially express any hematopoietic marker (CD235a, CD43, CD41a, and CD45) and maintained its endothelial identity expressing CD31, KDR, and CD144. In clonogenic assay or secondary culture, this third population showed a high proliferative potential and gave rise to multilineage erythroid and myeloid colonies as well as T lymphocytes. In addition, the generation of hematopoietic cells by this later population was more dependent on the presence of the DLL1 Notch ligand when compared with the other two populations. This observation is reminiscent of the differential Notch requirement observed during mouse embryonic development, where yolk sac hematopoiesis was found to be Notch independent, while intra-embryonic hematopoiesis was Notch dependent (Kumano et al., 2003). This suggests that the CD235a^+^CD31^−^ and CD235a^+^CD31^+^ populations could be the equivalents of the two yolk sac waves of hematopoiesis, primitive erythrocytes, and erythro-myeloid progenitors, described previously in the mouse embryo (McGrath et al., 2015). The CD235a^−^CD31^+^ population would represent the equivalent of intra-embryonic hematopoiesis, further supported by the definitive features displayed by this population including T lymphoid potential and the higher ratio of fetal versus embryonic globin. It is clear, however, that T cell potential on its own is not a hallmark of intra-embryonic hematopoiesis. Our data also suggest differences in lineage maturation from a pool of HE giving rise to the three CD31/CD235a populations with different hematopoietic potential, different proliferative potential, and different responsiveness to Notch. Together our findings demonstrate that HE present in EBs by day 6 of hESC differentiation already harbors primitive and definitive potential. An important question that remains to be addressed is whether different HE subsets give rise to the three CD31/CD235a populations or whether a unique HE generates all of them?

The derivation of hematopoietic stem and progenitor cells from hESCs usable in the clinic for therapeutic purpose remains a primary objective in the field of stem cell research and regenerative medicine. However, to achieve this goal, one first needs to have a perfect understanding of the differentiation process leading to the generation of these useful blood cells, both at the cellular and molecular level. Data presented here further our understanding of the developmental path of hESCs toward the generation of blood progenitor cells, bringing us a step closer to defining how these hematopoietic subsets are generated.

## Experimental Procedures

### hESC Culture and Differentiation

Man-5, Man-1 (University of Manchester), and H1 (WISC BANK USA) human ESCs were maintained on γ-irradiated CF1 (Millipore) or DR4 mouse embryonic fibroblasts in KO-DMEM (Gibco, Thermo Fisher Scientific) supplemented with 20% KO Serum replacement (Thermo Fisher Scientific), 0.1 mM 2-mercaptoethanol (50 mM, Gibco, Thermo Fisher Scientific), 1% minimum essential medium (MEM) non-essential amino acids solution (100×, Gibco, Thermo Fisher Scientific), 1% L-glutamine, 0.5% penicillin/streptomycin, and 8 ng/mL of human basic fibroblast growth factor (bFGF) (PeproTech). Four days before differentiation, hESCs were plated on Geltrex LDEV-Free, hESC-Qualified, reduced growth factor basement membrane matrix (Thermo Fisher Scientific) and maintained in TeSR-E8 (STEMCELL Technologies). Differentiation of hESC was driven by EB formation, using between 7 and 14 million hESCs, in serum-free StemPro-34 SFM (Thermo Fisher Scientific) supplemented with the sequential addition of human cytokines (all from PeproTech) during 6 days in low-oxygen conditions as follows: 10 ng/mL BMP4 at day 0, 5 ng/mL of bFGF at day 1, and 0.9 ng/mL activin A at day 2. From days 4 to 6 the medium was supplemented only with 5 ng/mL of bFGF and 12 ng/mL of VEGF. After 6 days, EBs were maintained in normoxia with or without the addition of hematopoietic cytokines as described previously (Kennedy et al., 2012). Similar and reproducible hematopoietic differentiation data were obtained from EB differentiation and HE culture using the three hESCs, Man-5, Man-1, and H1.

### OP9 Stromal Cell Lines Cultures

OP9-GFP carrying human DLL1 Notch ligand (Ayllon et al., 2015) and OP9-GFP were maintained in α-MEM basal medium (Thermo Fisher Scientific) supplemented with 20% heat-inactivated fetal bovine serum, 1% L-glutamine, and 1% penicillin/streptomycin. Both cell lines were γ-irradiated or treated with mitomycin C before co-culture.

### Purified HE Culture and Differentiation

Sorted CD31^+^CD144^+^ cells from EBs at the specified times were cultured on gelatin-coated plates or on co-culture with irradiated stroma cell lines. At day 6 of EB differentiation, the average percentage of sorted CD31^+^CD144^+^CD43^–^ HE population was 20%, resulting in the isolation of two to five million cells. The same numbers of sorted cells were seeded to analyze the hematopoietic differentiation across all culture conditions. Initial cell densities used in hematopoietic-inducing culture were as follows per well of a 6-well plate: 150,000 cells for days 2 and 3, 120,000 cells for day 4, and 75,000 for day 7. Hematopoietic differentiation from isolated CD31^+^CD144^+^ cells was performed in serum-free StemSpan medium (STEMCELL Technologies) supplemented with human cytokines (25 ng/mL insulin growth factor 1 (IGF-1), 25 ng/mL IGF-2, 50 ng/mL stem cell factor (SCF), 50 ng/mL thrombopoietin, 20 ng/mL Fms-like tyrosine kinase-3 ligand (FLT3L), 5 ng/mL interleukin-11 (IL-11), 5 ng/mL VEGF-A, and 5 ng/mL fibroblast growth factor 2 (all from PeproTech).

### T Lymphoid Differentiation

A total of 30,000 cells per well of a 12-well plate were cultured on mitomycin C-treated OP9-hDLL1 in stromal culture medium with human cytokines as follow: 20 ng/mL FLT3L, 100 ng/mL SCF, 25 ng/mL IL-2, and 5 ng/mL IL-7 during the first 2 weeks and 10 ng/mL FLT3L, 25 ng/mL IL-2, and 5 ng/mL of IL-7 for the last 2 weeks. The cultures were transferred to fresh OP9-hDLL1 cells every 5 days after the first 10 days. For passaging, the cultures were treated with TrypLe (Thermo Fisher Scientific) and filtered through a 50-μm sterile Filcon (BD). Cells were analyzed after 1 month of culture using hCD45-PeCy5.5 (eBioscience), anti-human CD8-PE (BioLegend), and anti-human CD4-PeCy7 (eBioscience).

## Author Contributions

E.G.-A. designed the research, performed the experiments, analyzed the data, and wrote the manuscript. S.M. contributed to the experiments. M.Z.H.F. performed the computational analyses. P.M. provided critical reagents. G.L. and V.K. designed the research, analyzed the data, and wrote the manuscript.

## Accession Numbers

RNA-seq data were deposited in the GEO under accession number GEO: GSE109648.

## Supplemental Information

Document S1. Figures S1–S7 and Table S1

Document S2. Article plus Supplemental Information
