# Supplementary material for: Early Human Hemogenic Endothelium Generates Primitive and Definitive Hematopoiesis In Vitro
Source: Stem Cell Reports. 2018 Oct 25;11(5):1061–74. doi: 10.1016/j.stemcr.2018.09.013 (PMC6234921; doi:10.1016/j.stemcr.2018.09.013)
Supplement: Document S1. Figures S1–S7 and Table S1 [file mmc1.pdf]

Stem Cell Reports, Volume *11*

Supplemental Information

Early Human Hemogenic Endothelium Generates Primitive and Definitive Hematopoiesis *In Vitro*

Eva Garcia-Alegria, Sara Menegatti, Muhammad Z.H. Fadlullah, Pablo Menendez, Georges Lacaud, and Valerie Kouskoff

SUPPLEMENTAL FIGURE AND LEGENDS

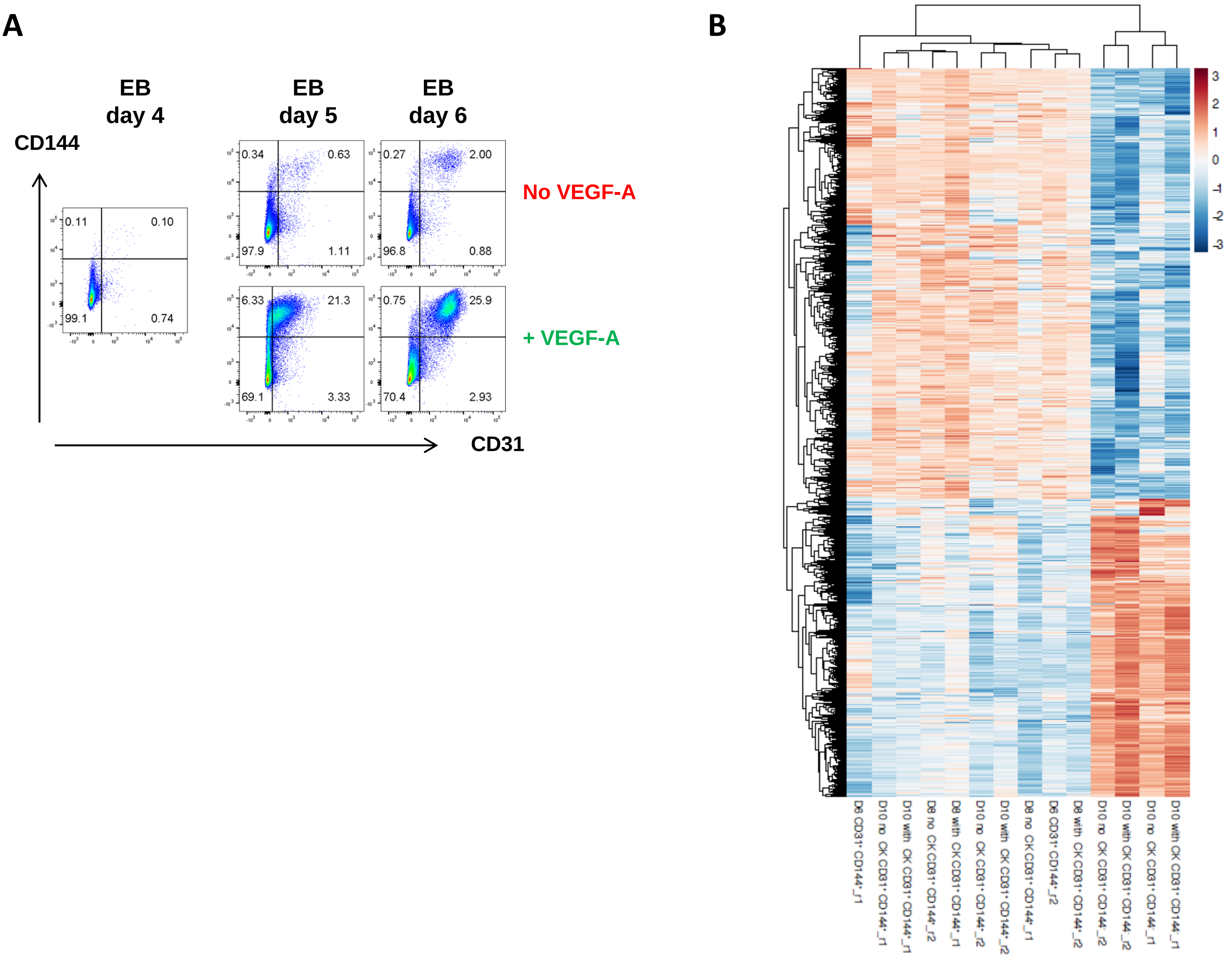

Figure S1: related to experimental procedures and Figure 1.

A. Effect of VEGF-A addition at day 4 of EB differentiation on the emergence of human $CD144^{+}CD31^{+}$ cell populations. Data shown are representative of 3 independent experiments.
B. Heat map of all differentially expressed genes (DEG) between the $CD31^{+}CD144^{+}$ population obtained at different times of EB differentiation and the hematopoietic committed population $CD31^{+}CD144^{neg}$ based on transcriptomic RNA-seq data.

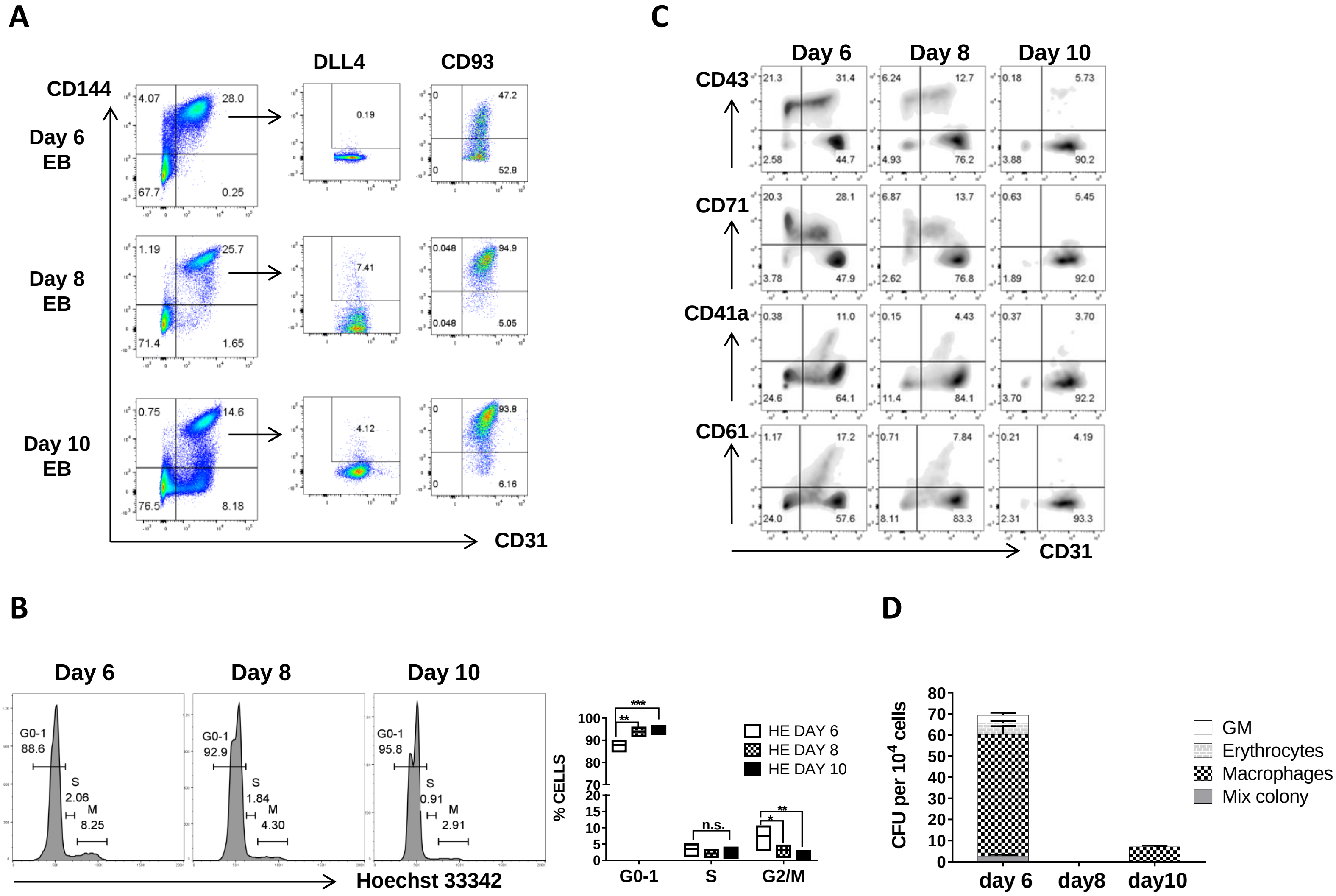

Figure S2: HE at different time points of the EB differentiation, related to figure 2

A. Flow cytometric analysis of DLL4 and CD93 expression within the CD31$^+$CD144$^+$ cell populations at day 6, 8 and 10 of embryoid body (EB) differentiation. Data shown are representative of 3 independent experiments.
B. Representative plot and statistical analysis of cell cycle of CD31$^+$CD144$^+$ cell populations isolated at the indicated times. Error bars indicate the SEM of data from 3 independent experiments. The significance of the difference between samples was confirmed using 2-way ANOVA; p-values *p< 0.05 ** p<0.001 ***p=0.0005.
C. Flow cytometric analysis of the hematopoietic profile obtained after 4 days of culture of CD31$^+$CD144$^+$CD43^{neg} isolated along the EB differentiation at the indicated times. Data shown are representative of 3 independent experiments.
D. Quantification of colony forming unit (CFU) potential of 104 cells obtained after 7 days of culture on gelatine-coated plates of CD31$^+$CD144$^+$ isolated from EBs at the indicated times. Error bars indicate the SEM of data from 3 independent experiments.

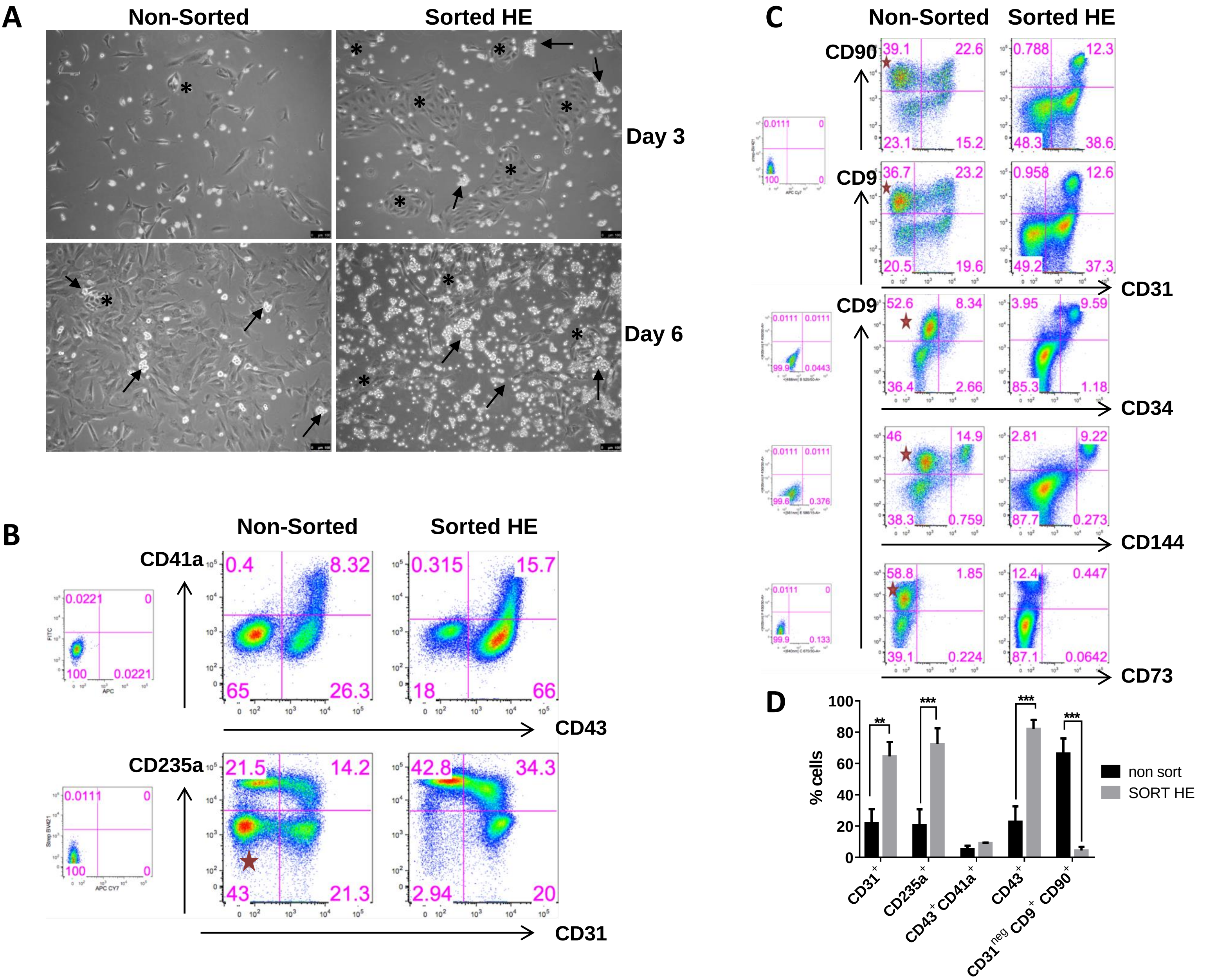

Figure S3: Enrichment of the HE, related to experimental procedures

A. Representative photographs of cell morphologies obtained after 3 and 6 days of culture on gelatin-coated dishes in hematopoietic-inducing condition from non-sorted day 6 embryoid body (EB) or from purified $CD31^{+}CD144^{+}$ cells at the same time of differentiation (scale bars 100µm). Progression and emergence of the hematopoietic round cells can be observed. Asterisks indicate endothelial clusters with hematopoietic cells emerging from them. Arrows indicate some of the emerging clusters of blood cells.

B. Flow cytometric analysis of the hematopoietic markers CD43, CD235a and CD41a and endothelial CD31 marker after 4 days of culture. Red star marks the presence of a cell population only present in the non-sorted culture (B-C).

C. Immuno-phenotypic characterization of the cell populations found in the non-sorted and sorted cultures after 4 days in gelatine coated culture. Data are representative of 3 independent experiments.

D. Quantification and statistical analysis of the flow cytometry data obtained after 4 days of culture. Error bars indicate the SEM of data from 3 independent experiments. The significance of the difference between samples was confirmed using 2-way ANOVA; p-value $**p=0.004$ and $***p<0.001$.

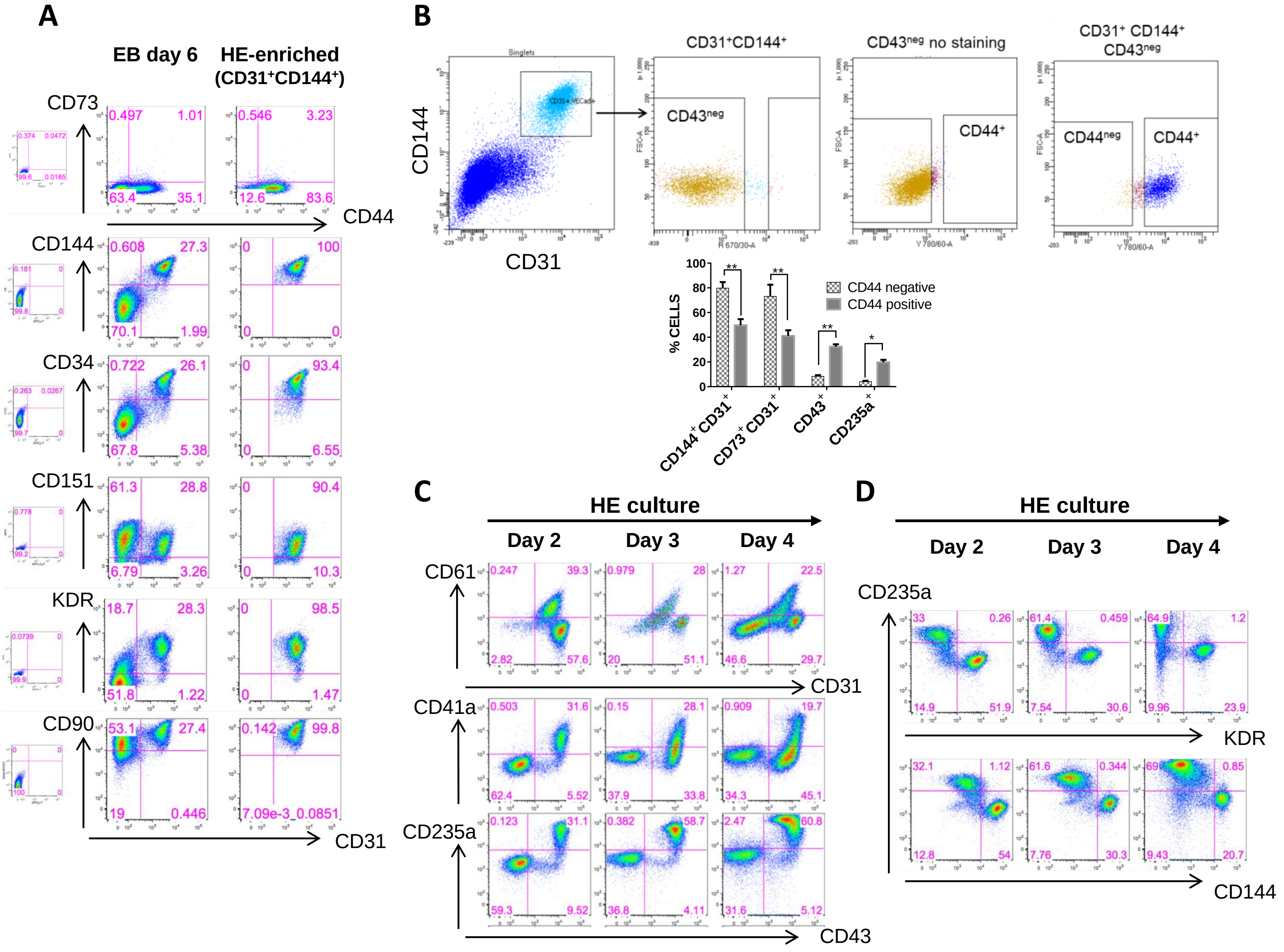

Figure S4: Purified HE of Man-5 cell line and hematopoietic development in gelatine, related to figure 3

A. Immuno-phenotypic characterization of $CD31^{+}CD144^{+}$ cells at day 6 of EB differentiation. Data shown are representative of 5 independent experiments.
B. Sorting strategy of $CD31^{+}CD144^{+}$ cells at day 6 of EB differentiation to better characterization of CD44 expressing population. Statistical analysis shows an increase of hematopoietic cells after 7 days in culture from the $CD44^{+}CD31^{+}CD144^{+}$ cell population. Error bars indicate the SEM of data from 3 independent experiments. The significance of the difference between samples was confirmed using 2-way ANOVA; p value $*p<0.05$ and $**p< 0.005$.
C. Flow cytometric analysis of the hematopoietic development obtained from sorted $CD31^{+}CD144^{+}CD43^{neg}$ on gelatine-coated plates at the times indicated. Data shown are representative of 3 independent experiments.
D. Downregulation of CD144 endothelial and KDR mesodermal marker in the hematopoietic population obtained from sorted $CD31^{+}CD144^{+}CD43^{neg}$ cultured on gelatine-coated plates and analysed at the indicated day of culture. Data shown are representative of 3 independent experiments.

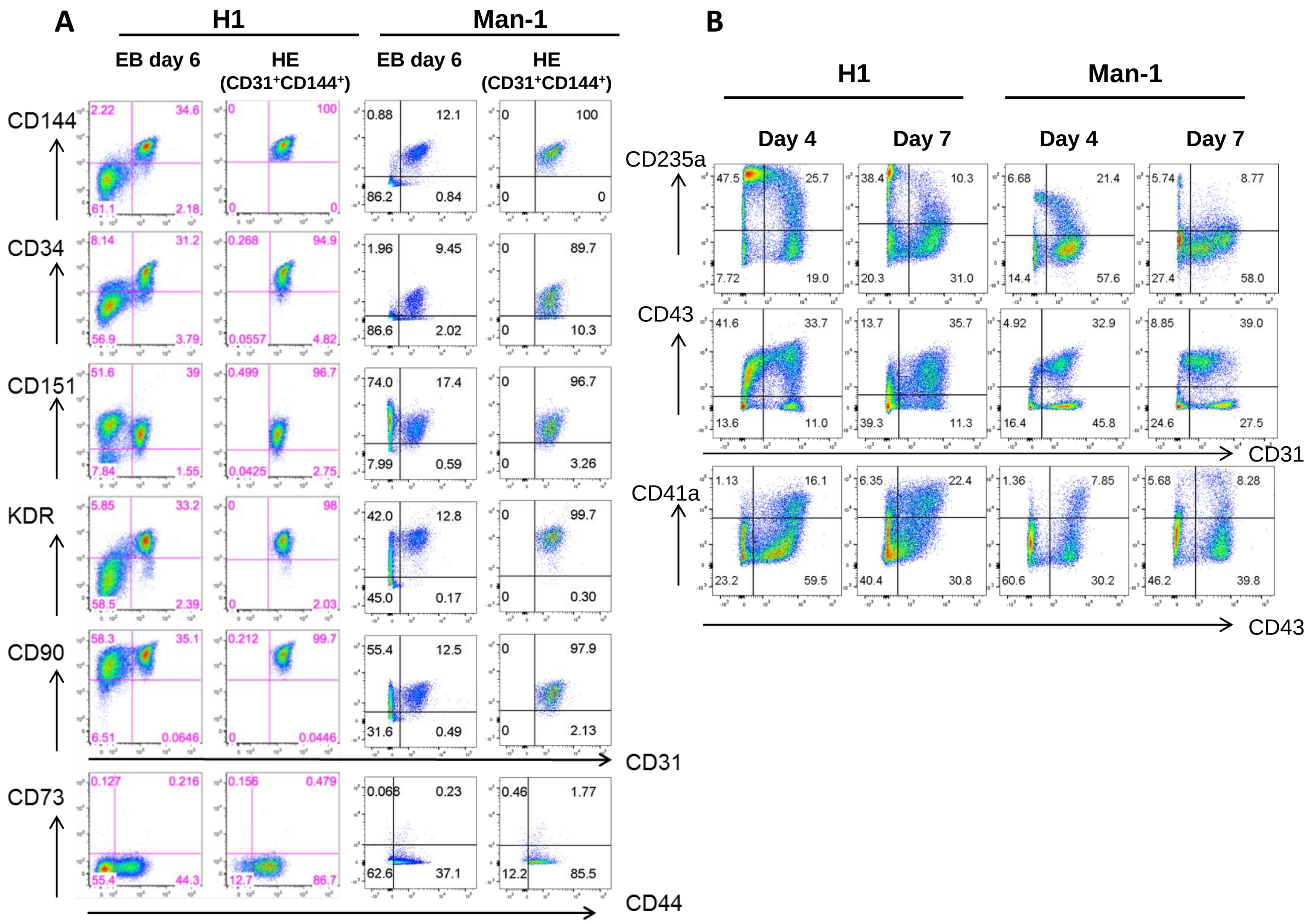

Figure S5: Characterization of HE and hematopoietic differentiation obtained from H1 and Man1 cell lines, related to experimental procedures.

A. Phenotypic characterization of $CD31^{+}CD144^{+}$ at day 6 of EB differentiation for H1 and Man1 hESC lines. Representative plots from 3 different experiments per cell line, showing reproducible marker characterization of the human HE-enriched populations.
B. Flow cytometric analysis of the hematopoietic development obtained from sorted $CD31^{+}CD144^{+}CD43^{neg}$ cells on gelatine-coated plates at day 4 and 7 for the H1 and Man1 hESC lines. Data shown are representative of 3 independent experiments per cell line.

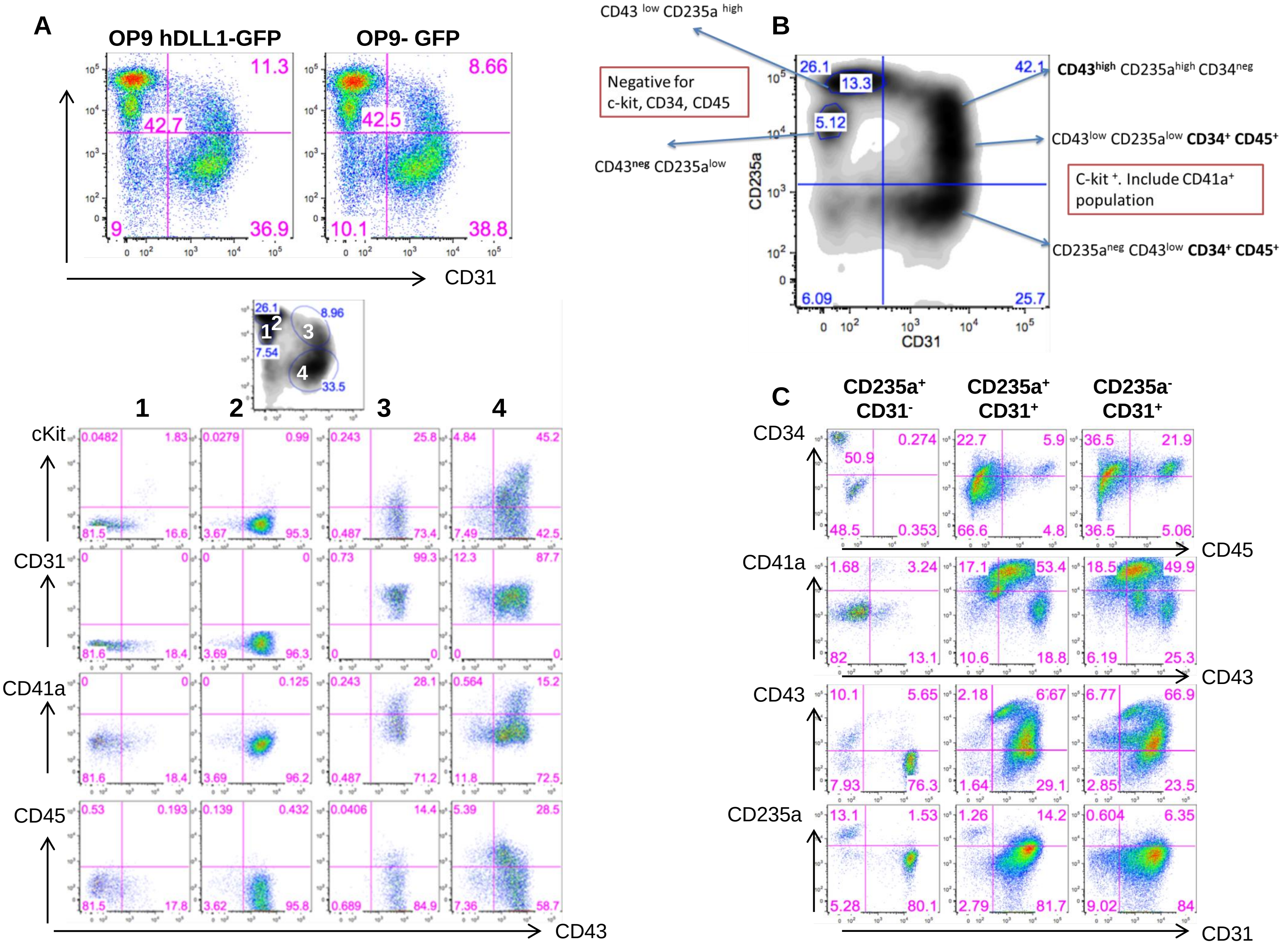

Figure S6: CD235a/CD31 kinetics in stromal co-culture, related to figure 3 and 5

A. Representative flow cytometric analysis of CD235a/CD31 populations obtained from $CD31^{+}CD144^{+}CD43^{neg}$ co-cultured for 7 days with irradiated OP9 and OP9-hDLL1 (upper panels). Characterization of the cell surface markers expressed or not in each population following 7 days of co-culture with stromal cells OP9-hDLL1, (lower panels). Data shown are representative of 3 independent experiments.
B. Summary of the cell surface markers expressed by each population after 7 days of $CD31^{+}CD144^{+}CD43^{neg}$ co-culture with stromal cells.
C. Flow cytometric analysis of hematopoietic development from the different CD235a/CD31 fractions after 7 day of culture on irradiated OP9 GFP stromal cells.

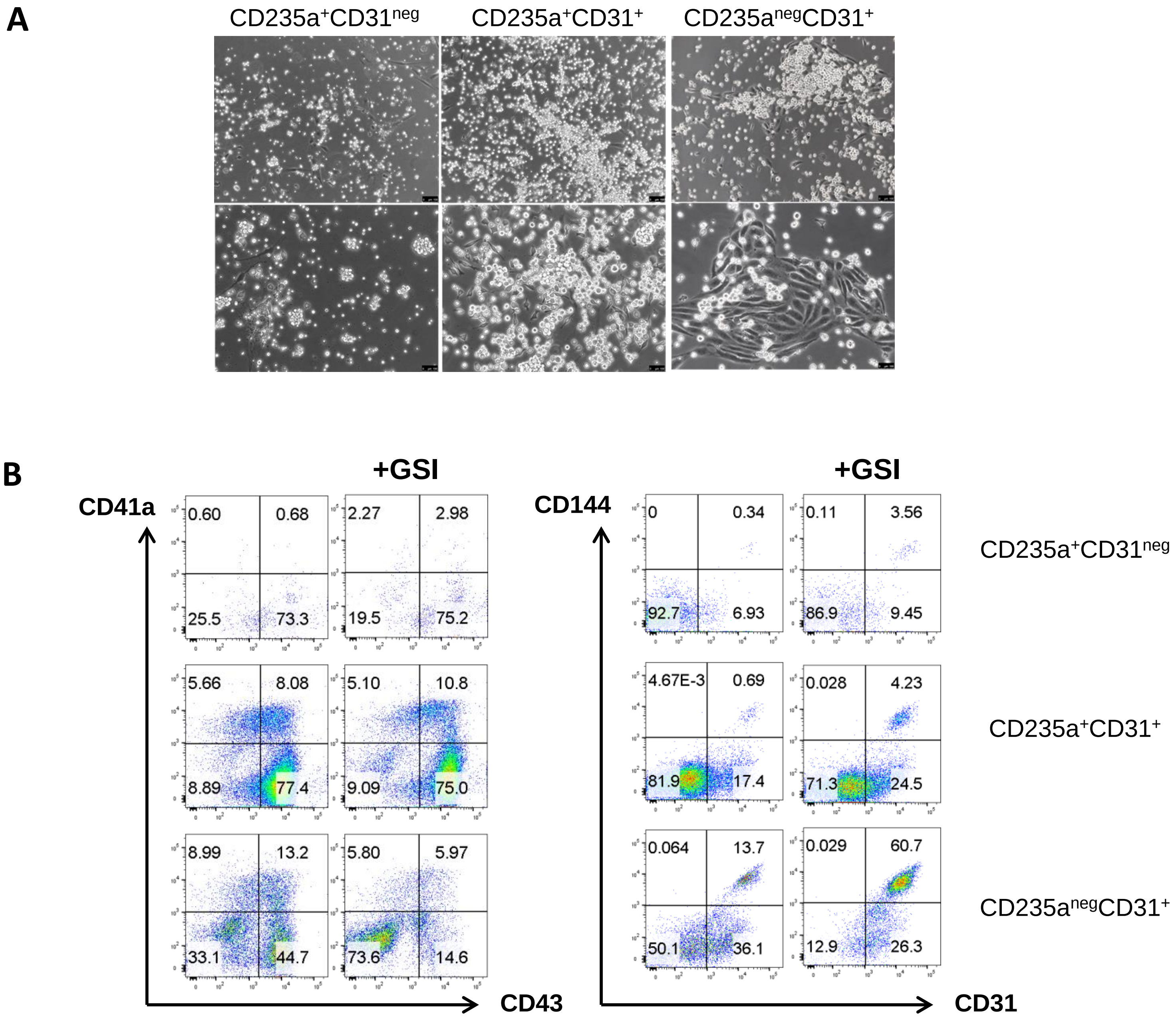

Figure S7: Culture of CD235a/CD31 populations in gelatine and effect of Notch inhibition in co-culture, related to figures 5 and 7.

A. Representative pictures of cell cultures obtained from sorted CD235a/CD31 fractions after 7 days of culture on gelatine-coated plates magnification (10X (upper row) and 20X (lower row)). Scale bars 100µm.
B. Effect of Notch inhibition as measured by the addition of 10 µM gamma secretase inhibitor (GSI) RO4929097 or DMSO control in the culture of the indicated populations after 7 days in co-culture with OP9h-DLL1. Data are representative of 3 independent experiments.

Table S1

EnsembleGeneID	symbol	Fold change	EnsembleGeneID	symbol	Fold change
ENSG00000135638	*EMX1*	0.032	ENSG00000139618	*BRCA2*	0.370
ENSG00000171388	*APLN*	0.038	ENSG00000161681	*SHANK1*	0.371
ENSG00000170373	*CST1*	0.052	ENSG00000163520	*FBLN2*	0.371
ENSG00000139438	*FAM222A*	0.070	ENSG00000139734	*DIAPH3*	0.372
ENSG00000115085	*ZAP70*	0.073	ENSG00000138821	*SLC39A8*	0.377
ENSG00000101441	*CST4*	0.076	ENSG00000134333	*LDHA*	0.377
ENSG00000109255	*NMU*	0.076	ENSG00000198838	*RYR3*	0.377
ENSG00000188803	*SHISA6*	0.096	ENSG00000184661	*CDCA2*	0.379
ENSG00000105672	*ETV2*	0.100	ENSG00000128683	*GAD1*	0.387
ENSG00000148704	*VAX1*	0.101	ENSG00000090530	*LEPREL1*	0.387
ENSG00000163492	*CCDC141*	0.119	ENSG00000115687	*PASK*	0.389
ENSG00000185666	*SYN3*	0.132	ENSG00000011426	*ANLN*	0.391
ENSG00000203805	*PPAPDC1A*	0.134	ENSG00000198826	*ARHGAP11A*	0.392
ENSG00000102385	*DRP2*	0.139	ENSG00000075218	*GTSE1*	0.395
ENSG00000155657	*TTN*	0.139	ENSG00000109805	*NCAPG*	0.398
ENSG00000075340	*ADD2*	0.142	ENSG00000186638	*KIF24*	0.400
ENSG00000197046	*SIGLEC15*	0.156	ENSG00000122966	*CIT*	0.405
ENSG00000143369	*ECM1*	0.164	ENSG00000131747	*TOP2A*	0.405
ENSG00000168280	*KIF5C*	0.164	ENSG00000050555	*LAMC3*	0.406
ENSG00000244588	*RAD21L1*	0.165	ENSG00000142945	*KIF2C*	0.414
ENSG00000222033	*LINC01124*	0.171	ENSG00000141526	*SLC16A3*	0.414
ENSG00000171643	*S100Z*	0.174	ENSG00000187164	*KIAA1598*	0.417
ENSG00000110092	*CCND1*	0.177	ENSG00000138778	*CENPE*	0.421
ENSG00000100351	*GRAP2*	0.182	ENSG00000166845	*C18orf54*	0.421
ENSG00000130477	*UNC13A*	0.193	ENSG00000139946	*PELI2*	0.424
ENSG00000166342	*NETO1*	0.197	ENSG00000101447	*FAM83D*	0.425
ENSG00000171587	*DSCAM*	0.198	ENSG00000178295	*GEN1*	0.427
ENSG00000158764	*ITLN2*	0.205	ENSG00000119969	*HELLS*	0.434
ENSG00000178568	*ERBB4*	0.235	ENSG00000079739	*PGM1*	0.436
ENSG00000159212	*CLIC6*	0.238	ENSG00000148773	*MKI67*	0.436
ENSG00000179715	*PCED1B*	0.249	ENSG00000126787	*DLGAP5*	0.440
ENSG00000105464	*GRIN2D*	0.262	ENSG00000123124	*WWP1*	0.441
ENSG00000159307	*SCUBE1*	0.263	ENSG00000162367	*TAL1*	0.442
ENSG00000095777	*MYO3A*	0.266	ENSG00000095637	*SORBS1*	0.444
ENSG00000189120	*SP6*	0.270	ENSG00000140534	*TICRR*	0.446
ENSG00000179403	*VWA1*	0.273	ENSG00000183850	*ZNF730*	0.447
ENSG00000111981	*ULBP1*	0.285	ENSG00000051341	*POLQ*	0.448
ENSG00000144136	*SLC20A1*	0.308	ENSG00000117724	*CENPF*	0.450
ENSG00000185565	*LSAMP*	0.310	ENSG00000180773	*SLC36A4*	0.450
ENSG00000186777	*ZNF732*	0.322	ENSG00000105227	*PRX*	0.450
ENSG00000166450	*PRTG*	0.334	ENSG00000137642	*SORL1*	0.451
ENSG00000155760	*FZD7*	0.337	ENSG00000162063	*CCNF*	0.454

ENSG00000123485	*HJURP*	0.338	ENSG00000166851	*PLK1*	0.456
ENSG00000233224	*HIST1H2AM*	0.339	ENSG00000138182	*KIF20B*	0.460
ENSG00000066279	*ASPM*	0.344	ENSG00000161800	*RACGAP1*	0.461
ENSG00000159399	*HK2*	0.345	ENSG00000100629	*CEP128*	0.463
ENSG00000135476	*ESPL1*	0.355	ENSG00000146263	*MMS22L*	0.464
ENSG00000167703	*SLC43A2*	0.357	ENSG00000131389	*SLC6A6*	0.472
ENSG00000088826	*SMOX*	0.358	ENSG00000060982	*BCAT1*	0.472
ENSG00000168421	*RHOH*	0.363	ENSG00000068489	*PRR11*	0.473
ENSG00000137812	*CASC5*	0.369			

EnsembleGeneID	symbol	Fold change	EnsembleGeneID	symbol	Fold change
ENSG00000150681	*RGS18*	489.068	ENSG00000168938	*PPIC*	3.821
ENSG00000176956	*LY6H*	234.933	ENSG00000143603	*KCNN3*	3.819
ENSG00000166979	*EVA1C*	109.390	ENSG00000112149	*CD83*	3.817
ENSG00000133800	*LYVE1*	109.316	ENSG00000005108	*THSD7A*	3.772
ENSG00000188536	*HBA2*	86.679	ENSG00000137266	*SLC22A23*	3.763
ENSG00000145192	*AHSG*	66.773	ENSG00000132357	*CARD6*	3.630
ENSG00000000971	*CFH*	39.367	ENSG00000111261	*MANSC1*	3.629
ENSG00000079385	*CEACAM1*	35.535	ENSG00000205336	*GPR56*	3.596
ENSG00000099260	*PALMD*	17.622	ENSG00000182240	*BACE2*	3.491
ENSG00000150630	*VEGFC*	17.299	ENSG00000169594	*BNC1*	3.332
ENSG00000118777	*ABCG2*	15.342	ENSG00000071242	*RPS6KA2*	3.201
ENSG00000176435	*CLEC14A*	11.762	ENSG00000075426	*FOSL2*	3.184
ENSG00000182851	*GPIHBP1*	11.583	ENSG00000104870	*FCGRT*	3.165
ENSG00000113389	*NPR3*	10.919	ENSG00000173598	*NUDT4*	3.108
ENSG00000081051	*AFP*	10.575	ENSG00000240583	*AQP1*	3.107
ENSG00000101542	*CDH20*	10.468	ENSG00000163171	*CDC42EP3*	3.088
ENSG00000146674	*IGFBP3*	10.344	ENSG00000001561	*ENPP4*	3.049
ENSG00000172031	*EPHX4*	9.580	ENSG00000145623	*OSMR*	3.027
ENSG00000129422	*MTUS1*	9.079	ENSG00000164930	*FZD6*	2.892
ENSG00000132872	*SYT4*	7.611	ENSG00000185112	*FAM43A*	2.876
ENSG00000175874	*CREG2*	7.029	ENSG00000164035	*EMCN*	2.871
ENSG00000154133	*ROBO4*	6.793	ENSG00000147862	*NFIB*	2.863
ENSG00000171864	*PRND*	6.676	ENSG00000143801	*PSEN2*	2.840
ENSG00000136960	*ENPP2*	6.575	ENSG00000166750	*SLFN5*	2.707
ENSG00000184113	*CLDN5*	6.308	ENSG00000197461	*PDGFA*	2.679
ENSG00000116016	*EPAS1*	6.018	ENSG00000176597	*B3GNT5*	2.539
ENSG00000143140	*GJA5*	5.109	ENSG00000069122	*GPR116*	2.476
ENSG00000137393	*RNF144B*	4.982	ENSG00000198168	*SVIP*	2.428
ENSG00000127241	*MASP1*	4.728	ENSG00000078269	*SYNJ2*	2.401
ENSG00000213949	*ITGA1*	4.712	ENSG00000164929	*BAALC*	2.311
ENSG00000179144	*GIMAP7*	4.678	ENSG00000125810	*CD93*	2.254
ENSG00000165702	*GFI1B*	4.430	ENSG00000123240	*OPTN*	2.249
ENSG00000167680	*SEMA6B*	4.085	ENSG00000126785	*RHOJ*	2.134
ENSG00000133574	*GIMAP4*	4.063	ENSG00000101974	*ATP11C*	2.108
ENSG00000165507	*C10orf10*	4.035	ENSG00000170989	*S1PR1*	2.096
ENSG00000124772	*CPNE5*	3.929			

Table S1: List of the genes down-regulated (Blue) and up-regulated (red) between day 6 and day 10 in the CD31$^+$CD144$^+$ populations sorted from embryoid body EB differentiation. Related to Figure 1. Differentially expressed genes are derived from RNA-seq data analysis. The data were filtered to include only genes with at least 1 count-per-million reads and genes with a median coverage of more than 10%. Genes with > 2-fold change and false discovery rate < 0.05 were considered as differentially expressed.

SUPPLEMENTAL EXPERIMENTAL PROCEDURES

Flow cytometry and cell sorting: For HE-enriched population sorting, cells were stained with hCD144-PE (Miltenyi), hCD43-APC (eBioscience) and hCD31-APC-Cy7 (eBioscience). Further characterization of human HE at day 6 of EB differentiation was performed using hCD90-Biotin (eBioscience), hCD44-PeCy7 (eBioscience), hCD34-FITC (Miltenyi), hCD117-PeCy7 (eBioscience), hKDR-AlexaFluor-647 (eBioscience), hCD151-APC (eBioscience), hCD9-Biotin (eBioscience), hCD73-APC (eBioscience), hDLL4-Biotin (Miltenyi) and hCXCR4-PeCy5.5 (eBioscience). Analysis of hematopoietic differentiation was assessed using hCD41a-FITC (eBioscience), hCD45-PeCy5.5 (eBioscience), hCD61-FITC (Miltenyi) CD71-FITC (eBioscience), and hCD235a-Biotin (eBioscience). BV421-Streptavidin (Biolegend) was used for all biotinylated antibodies. Non-viable cells were excluded by Hoechst 33258 staining. When co-culture was used the stromal cells were excluded based on their size and on their high expression level of GFP expression. Compensations were performed with beads controls. Acquisition was performed on LSRII or Fortessa (BD Biosciences) and data were analysed using FlowJo software (Treestar). Cell sorting was performed on Aria III or Influx sorters (BD Biosciences).

Clonogenic assay: Hematopoietic potential was assessed in semi-solid methylcellulose culture. As specified on each graphs, 7 to 10 $x10^3$ cells were seeded into IMDM media (Thermo Fisher) containing 1% methylcellulose and human cytokines as previously described (Kennedy et al., 2012). Counting was performed after 14 days of culture at 37ºC in 5%CO^2. All assays were performed in triplicate.

Cell cycle: CD31+CD144+CD43neg cells were isolated at day 6, 8 and 10 of EB differentiation. 30,000 unfixed sorted cells maintained in 500 µl of Stemspan medium (Stem Cell Technologies) were stained by incubation with 5µl of 50µM Hoechst 33342 (Thermo Fisher) for 30 min at 37°C. For live discrimination 5 µl 7-amino actinomycin-D (7-AAD) (Thermo Fisher) was added 5 min before analysis at room temperature.

Globin expression: RNA was extracted from hematopoietic colonies obtained after 2 weeks in methylcellulose culture from each population identified by CD235a/CD31 markers. Assessment of globin expression was performed by real time PCR using the StepOne plus system (Thermo Fisher) and the human assays Hs00362216_m1 HBE1 Hs00361131_g1 HBG1/2 and the Taqman universal master mix II (Thermo Fisher). Comparative Ct Method was used for relative quantification of the RNA expression using as housekeeping gene the human assay Hs00187842_m1 B2M (Thermo Fisher). Quantification and statistical analysis were performed from 3 independent experiments containing 3 replicates of the CFU culture per experiment and population. Statistical significance was assessed by 2-way ANOVA.

Notch pathway inhibition: The three CD235a/CD31 populations were isolated from day 4 of HE culture and 30,000 cells of each population per well of a 12-well plate were further cultured on OP9-hDLL1 stroma cells with the addition of 10µM of gamma secretase inhibitor RO4929097 or control DMSO for 7 days prior to analysis.

RNA extraction and cDNA synthesis: Total RNA was extracted using RNeasy Mini Kit (Qiagen). cDNA was obtained with GoScript™ Reverse Transcriptase mix (Promega).

RNA-Seq Libraries: Indexed total RNA libraries were prepared using 10ng of Total RNA, a 4-minute fragmentation time, and 12 cycles of amplification in the SMARTer Stranded Total RNA-Seq Kit-Pico input (Clontech). Libraries were quantified by qPCR using a Kapa Library Quantification Kit for Illumina sequencing platform (Kapa Biosystems Inc. Cat No: KK4835). Single end 75bp sequencing was carried out by clustering 1.8 pM of the pooled libraries on a NextSeq 500 sequencer (Illumina Inc.).

RNA-sequencing data analysis: Basecall files generated from HiSeq sequencing run were converted to FASTQ format with Illumina's bcl2fastq. Lane-wise alignment was performed by bowtie2 (version 2.2.1) (Langmead and Salzberg, 2012) to human reference genome (GRCh37.75) with default parameters. Generated SAM files from bowtie2 alignment were converted to BAM files by samtools v0.1.19. Parameters for samtools SAM to BAM conversion: -q 10 -f 2 -F 260. Resulting lane-wise BAM files from the same sequence library was merged into one BAM file used for downstream analysis. The expression levels of 57,773 annotated features were determined by using the featureCounts (Liao et al., 2014) function from the Bioconductor package Rsubread (version 1.13.13). The Bioconductor package edgeR (Robinson et al., 2010) (version 3.8.5) was used to identify genes that showed statistically significant variation in expression levels. The data was filtered to include only genes with at least 1 count-per-million reads and genes with a median coverage of more than 10%. Differential expression analysis was performed using the function exactTest in edgeR (Robinson et al., 2010). Genes with > 2 fold change and false discovery rate < 0.05 were considered as differentially expressed. Gene ontology analysis of the DEG was performed using DAVID on line tool.

Statistical Analyses: Statistical analyses were performed using Graph Pad Prism Version7.0 (Graph Software). Error bars indicate the SEM of data from independent experiments. Statistical differences were assessed by 2-way ANOVA using SIDAK´s or Turkey multiple comparison test to obtain the multiplicity adjusted p-values as showed in the graphs and legends.

Mitotic inactivation of OP9 stroma cells by Mitomycin C. Stroma cells thawed in a 162 cm2 flask were maintained in alpha MEM 20% FBS (as described in experimental procedures) until 90% confluence is reached. The cells were then split in 4 flasks and once they reached 90% confluence they were treated with fresh media containing 10µg/ml of Mitomycin C (Bio Techne) for 3 hours. Cultures were then washed twice with media and twice with PBS to remove any trace of the drug. After the treatment cells were trypsinized, counted and frozen at -80C in a density of 1 million cells per vial, normally used within 2 months.

SUPPLEMENTAL REFERENCES

Langmead, B., and Salzberg, S.L. (2012). Fast gapped-read alignment with Bowtie 2. Nat Methods 9, 357-359.

Liao, Y., Smyth, G.K., and Shi, W. (2014). featureCounts: an efficient general purpose program for assigning sequence reads to genomic features. Bioinformatics 30, 923-930.

Robinson, M.D., McCarthy, D.J., and Smyth, G.K. (2010). edgeR: a Bioconductor package for differential expression analysis of digital gene expression data. Bioinformatics 26, 139-140.
